# Concomitant Photoresponsive Chiroptics and Magnetism in Metal-Organic Frameworks at Room Temperature

**DOI:** 10.34133/2021/5490482

**Published:** 2021-02-10

**Authors:** Bin Xia, Qian Gao, Zhen-Peng Hu, Qing-Lun Wang, Xue-Wei Cao, Wei Li, You Song, Xian-He Bu

**Affiliations:** ^1^College of Chemistry, State Key Lab of Elemento-Organic Chemistry, Nankai University, Tianjin 300071, China; ^2^School of Physics, Nankai University, Tianjin 300071, China; ^3^School of Materials Science and Engineering, Tianjin Key Lab of Metal and Molecule-Based Material Chemistry, Nankai University, Tianjin 300350, China; ^4^State Key Lab of Coordination Chemistry, School of Chemistry and Chemical Engineering, Nanjing University, Nanjing 210023, China

## Abstract

Stimulus-responsive metal-organic frameworks (MOFs) can be used for designing smart materials. Herein, we report a family of rationally designed MOFs which exhibit photoresponsive chiroptical and magnetic properties at room temperature. In this design, two specific nonphotochromic ligands are selected to construct enantiomeric MOFs, {Cu_2_(L-mal)_2_(bpy)_2_(H_2_O)·3H_2_O}_n_ (1) and {Cu_2_(D-mal)_2_(bpy)_2_(H_2_O)·3H_2_O}_n_ (2) (mal = malate, bpy = 4, 4′ − bipyridine), which can alter their color, magnetism, and chiroptics concurrently in response to light. Upon UV or visible light irradiation, long-lived bpy^−^ radicals are generated via photoinduced electron transfer (PET) from oxygen atoms of carboxylates and hydroxyl of malates to bpy ligands, giving rise to a 23.7% increase of magnetic susceptibility at room temperature. The participation of the chromophores (-OH and -COO^−^) bound with the chiral carbon during the electron transfer process results in a small dipolar transition; thus, the Cotton effects of the enantiomers are weakened along with a photoinduced color change. This work demonstrates that the simultaneous responses of chirality, optics, and magnetism can be achieved in a single compound at room temperature and may open up a new pathway for designing chiral stimuli-responsive materials.

## 1. Introduction

Stimulus responsive metal-organic frameworks (MOFs) which can alter their physical properties upon external stimuli are promising candidates for the design of smart functional materials. In particular, photoresponsive MOFs have great advantages in a range of applications such as sensors, electrooptic devices, and molecular switches due to their high controllability and the instantaneity of light [1–8]. Among them, photochromism and photomagnetism are two important kinds of behaviors. The former can feature a sudden change of color as well as some physical properties in response to light, while the latter leads to variations of magnetic properties. Photochromic compounds are potential candidates for the creation of optically controllable coloring devices without extra detectors [9–14]. Photomagnetism, especially with switchable magnetic behavior in molecular magnets, is of great interest since it offers promising application potential in high-density information storage and quantum computing. The combination of photochromic and photomagnetic behavior offers fascinating opportunities in smart material systems [15–17].

Over the last two decades, a great deal of photomagnetic compounds was reported which can mainly be ascribed to two classes: light-induced excited spin-state trapping (LIESST) compounds and cyano-bridged spin transition compounds such as Prussian blue analogues (PBAs) [18]. Photomagnetism of the former results in the variation of diverse electron configurations while photoinduced metal-metal charge transfer (MMCT) occurs in the latter [19, 20]. However, both classes of photomagnetic compounds can exhibit transformable magnetic behavior only at low temperature, which strictly limits their future applications [21]. In 2015, photomagnetism was reported at room temperature (RT) in a PBA compound [Eu(18-Crown-6)(H_2_O)_3_][FeCN_6_] attributing to a photoinduced intramolecular redox reaction between Fe(III) cations and crown ether ligands [22]. Meanwhile, a new kind of photochromism was discovered in metal-assisted electron transfer systems, namely metalloviologen [23–26]. In these systems, oxygen- and nitrogen-containing ligands serve as electron donors and acceptors, respectively. When a suitable electron transfer pathway exists and the relevant energy levels match, long-lived photoinduced charge-separated (CS) states can be generated under illumination. By construction of such a D-M-A coordination system, photoinduced electron transfer (PET) and RT photomagnetism can coexist. Benefiting from the structural designability of MOFs, integrating chiral and photoresponsive units in MOFs would bring new functionalities to such D-M-A coordination systems [27].

**Scheme 1:**
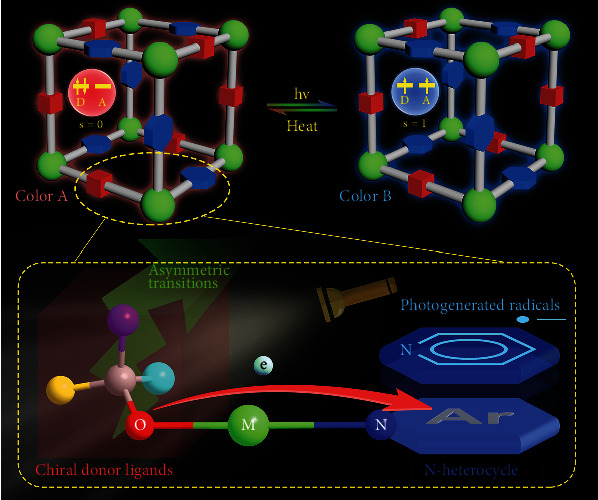
Photoinduced electron transfer of chiral metalloviologen.

In this work, the assembly of a D-M-A system was elaborately designed (Scheme 1). In consideration of magnetic coupling effects, monodentate ligands and diamagnetic metal cations are excluded. 4,4′-Bipyridine is chosen as the acceptor ligand which is a basic acceptor in the metalloviologen model system. In the selection of donor ligands, one of the simplest chiral dicarboxylic acids, malic acid, was chosen as a model ligand due to its large lone pair numbers and good coordination ability. The Jahn-Teller effect associated with Cu(II) ions can usually result in solvent coordination at a vertical axis or square coordination modes to produce a relatively strong coordination bonding between the metal and target ligands (donor or acceptor ligands); this enhances the possibility of metal-assisted electron transfer between donors and acceptors. These rational insights led to this report of three Cu(II) MOFs by the construction of a D-M-A system. By using chiral and racemic reagents in the synthesis, respectively, two chiral MOFs, {Cu_2_(L-mal)_2_(bpy)_2_(H_2_O)·3H_2_O}_n_ (1) and {Cu_2_(D-mal)_2_(bpy)_2_(H_2_O)·3H_2_O}_n_ (2), and a racemic analogue {Cu(DL-mal)(bpy)}_n_ (3) were obtained. In the enantiomeric compounds, a long-lived CS state was enabled via electron transfers from O atoms of carboxylate and hydroxy of malate to bpy rings upon irradiation. Apart from color change, the isomerization between ground states (GS) and CS states shows a distinct magnetic behavior at room temperature. Moreover, the participation of chiral components in the PET process produces a very rare chiroptical behavior in MOF systems.

## 2. Results

### 2.1. Crystal Structure

**Figure 1:**
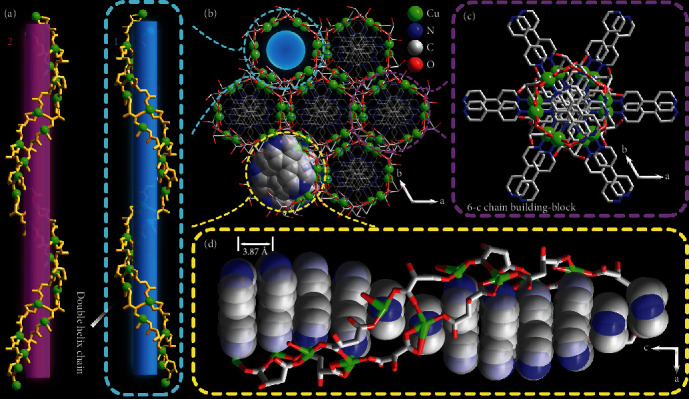
Structure of 1. H atoms and free water molecules in the framework are omitted for clarity. (a) Double helical [Cu_2_(mal)_2_(H_2_O)]_n_ chains in 1 and 2. (b) Projection of the 3D framework of 1 at the *ab* plane. Crosslinking bpy ligands penetrating the double-helical chain viewed at *ab* (c) and *ac* (d) plane.

The structure refinement details and selected bond lengths and angles are given in Table S1 and Table S2, respectively. Single crystal X-ray diffraction analysis revealed that 1 crystallizes in the hexagonal space group *P*6_1_ (No. 169) and presents a 3D chiral framework structure [28]. As shown in Figure S1, the asymmetric unit of 1 consists of two crystallographically independent Cu atoms (Cu1, Cu2), two bpy ligands, two malate ions, one coordinated water molecule, and three unbonded water molecules. Each Cu1 center is ligated by three carboxylic oxygens (O1, O6, O9) and two bipyridyl nitrogens (N1, N2) in a [CuN_2_O_3_]-distorted tetragonal-pyramidal geometry. Cu2 atom is also five coordinated and lies in a tetragonal-pyramidal coordination geometry. The water molecule (O11) is coordinated with a Cu2 atom and located at the Jahn-Teller axis. Malate ions adopt two kinds of coordination modes connecting the adjacent copper atoms to construct a right-handed DNA-like double-stranded helical chain with components of [Cu_2_(L-mal)_2_(H_2_O)]n along the c axis (Figure 1(a)). The neighboring chains are further linked via bpy in the ab plane (Figure 1(b)–1(d)) to constitute a 3D framework, and 1D-chiral channels are formed and filled with water molecules along the *c* axis (Figure S1). By the assembly of Cu1 sites as five-connected (5-c) nodes, Cu2 sites as 4-c nodes, and malate (C25-C28) as 3-c nodes, 1 exhibits a topology of Schafoli symbol {5·6^2^·7^2^·8}{5^2^·6^2^·7^5^·8}{5^2^·8} (Figure S1).

The left-handed 2 crystallizes in the hexagonal space group *P*6_5_ (No. 170) and is the enantiomer of compound 1 in structure (Figure 1(a)) with opposite screw direction in the framework. When using racemic malic acid as starting reagents in synthesis, Complex 3 can be found in a mixture of blue crystals (Figure S2). Diffraction data revealed that 3 exhibits a 3D-network with an unreported topology {4·6^2^}^2^{4^2^·6^8^·8^3^·10^2^}{6^4^·8^2^} (td10 = 1170) (Figure S3).

### 2.2. Photochromism

**Figure 2:**
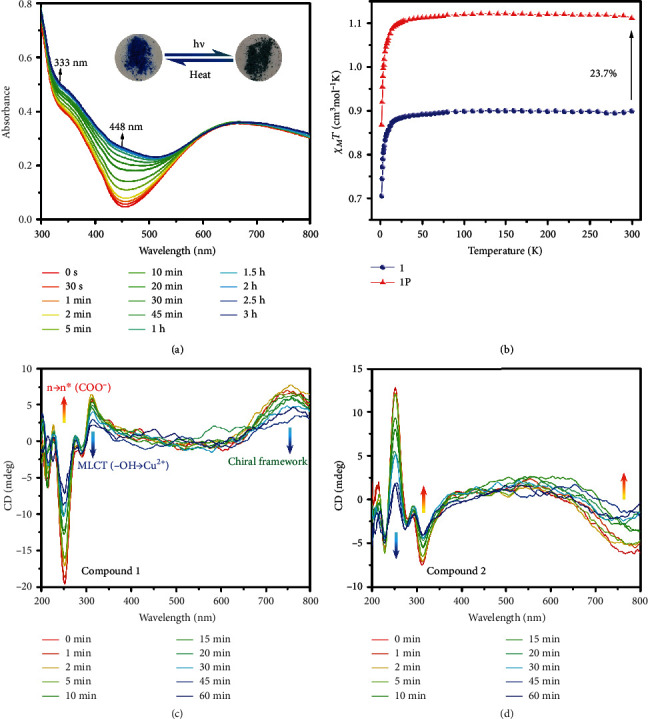
(a) Time-dependent diffuse-reflectance UV-vis absorption spectra and photographs of 1 upon UV light irradiation. (b) Plots of *χ*_*M*_ and *χ*_*M*_*T* versus *T* for 1 (○) and 1P (△). Time-dependent solid-state CD spectra of 1 (c) and 2 (d) at room temperature upon irradiation.

Both 1 and 2 can exhibit photochromic behavior. Photochromism of 1 and 2 can be triggered by both UV and visible light irradiation of xenon lamp (Figure S4). As shown in Figure 2(a), upon UV irradiation for an hour, the colors of compounds 1 and 2 change from blue to dark cyan. To further explore the photosensitivity of photochromism, a wavelength-dependent study was performed. As shown in Figure S5, fresh samples can change their color upon irradiation for 15 min with light (*λ* ≤ 400 nm) and are most sensitive to light with *λ* = 350 nm. The colorization and decolorization process of 1 and 2 are revisible. The irradiated samples can generally return to their original blue color upon heating in the existence of water or set at ambient conditions in the dark, indicating their typical feature of T type photochromic species. Time-dependent diffuse-reflectance UV-vis spectra measurements of compound 1 show a shoulder absorption peak around 350 nm which mainly arises from charge transfer from the alcoholate chromophore to Cu(II) ions and part of *n* → *π*^∗^ transition of bipyridine while the broad absorption band around 670 nm can be assigned to the d-d transitions of square-based pyramidal geometry (*C*_4v_) of Cu(II) centers. The absorbance of 1 increases with increasing UV irradiation time and the absorption changes become saturated after two hours. Two new bands at around 333 and 448 nm appeared after UV irradiation.

### 2.3. Magnetism and Photomagnetism

**Figure 3:**
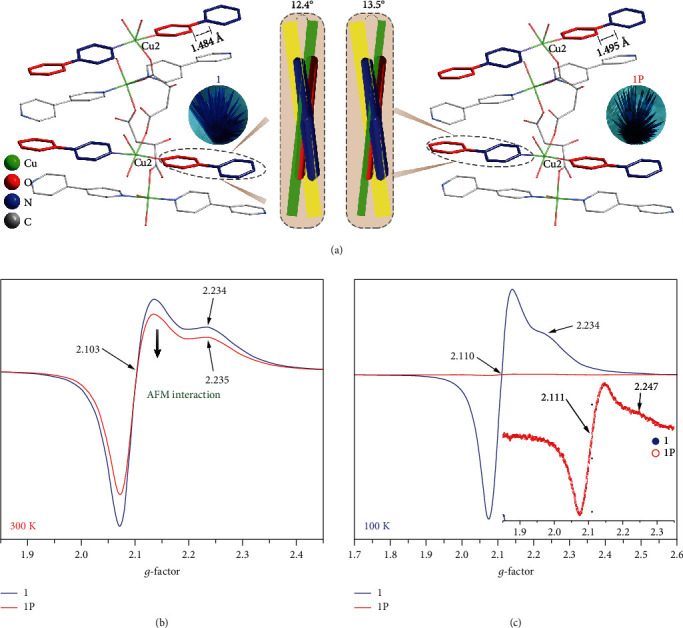
(a) Structure difference between 1 and 1P at 100 K. The solid-state EPR spectra of 1 and 1P at 300 K (b) and 100 K (c). Insert: enlarged sized EPR signal of 1P at 100 K.

The magnetic susceptibilities of compounds 1 and 1P over 2–300 K were measured under 1000 Oe. The *χ*_*M*_*T* value of 1 is 0.900 cm^3^ mol^−1^ K at room temperature (Figure 2(b)). The experimental value is much higher than the spin-only value (0.75 cm^3^ mol^−1^ K) for two Cu(II) ions (*S* = 1/2, *g* = 2). However, in consideration of the anisotropy of *g*-factors for Cu(II) compounds, the value is close to that (0.868 cm^3^ mol^−1^ K) calculated by the result of EPR measurement *g* = 2.148 (*g*^2^ = (*g*_||_^2^ + 2*g*_⊥_^2^)/3) (Figure 3(b)). As temperature decreased from 300 to 25 K, the *χ*_*M*_*T* value stays essentially constant. Upon further cooling, the*χ*_*M*_*T*values firstly decrease slightly and then rapidly reduce, reaching 0.706 cm^3^ mol^−1^ K at 2 K. The Curie–Weiss fitting gives *θ* = −0.37 K and *C* = 0.90 cm^3^mol^−1^K, indicating major antiferromagnetic interactions existing between adjacent Cu(II) ions (Figure S6). The magnetic analysis of 1 was carried out by using the spin Hamiltonian $\hat{H}=-2J{\hat{S}}_{1}{\hat{S}}_{2}$ to deal with the interactions through the carboxylate bridge between two neighboring Cu(II) ions, and intermolecular interactions (*zJ*′) can be used to deal with the magnetic interactions between the binuclear units in the molecular field approximation. So the resulting magnetic susceptibility equation is

(1) $$\begin{array}{c} \chi_{M}=\frac{2Ng^{2}\beta^{2}}{KT}\left[\frac{1}{3+\exp\left(-2J/KT\right)}\right]+N_{\alpha },\\ {\chi_{M}}^{\prime }=\frac{\chi_{M}}{1-\left(2zj^{\prime }/{Ng}^{2}\beta^{2}\right)\chi_{M}} \end{array}$$

(*N*_*α*_ = 120 × 10^−6^ cm^3^mol^−1^). *J* is the intramolecular exchange integral between Cu(II) ions through the carboxylate bridge; the other symbols have their usual meanings. The best fitting for the experimental data gives *J* = −0.432 cm^−1^, *g* = 2.19, and *zJ*′ = −7.61 × 10^−2^ cm^−1^. The agreement factor *R* = ∑(*χ*_obsd_ − *χ*_cacld_)^2^/∑*χ*_obsd_^2^ is 3.21 × 10^−6^ (Figure S7). With an increasing external magnetic field, an isothermal magnetization *M*(*H*) curve at 2 K of 1 shows a linear increase before 2 T, then gradually increases to a saturation value of 2.10 *Nμ_B_* after 5.5 T (Figure S8).

After irradiation, the *χ*_*M*_*T* value (1.113 cm^3^ mol^−1^ K) of 1P at room temperature was 23.7% higher than that of compound 1 (Figure 2(b)). Upon cooling, the *χ*_*M*_*T* value of 1P shows a similar behavior as compound 1 and reaches the values of 0.869 cm^3^ mol^−1^ K at 2 K. As for 1P, since the position and the quantity of the radicals cannot be defined in all lattice, a general Curie-Weiss fitting was conducted as a result of *C* = 1.12 cm^3^mol^−1^K, *θ* = −0.34 K (Figure S6). The similar value between *C* and *χ*_*M*_*T* at room temperature of irradiated samples and the negative value of *θ* suggest a possible antiferromagnetic interaction exists between two Cu(II) ions and radicals. The *M-H* curve of 1P shows a monotonous increasing trend with the field and has a value of 2.64 *Nμ_B_* for its final value at 7 T (Figure S8).

### 2.4. Chiroptics

CD spectra of 1 and 2 indicate that they are enantiomers (Figure S9). The part before 360 nm of the CD spectra of 1 matches well with the experimental results of copper L-malate aqua solution, which arises from the chiral malate ligands [29]. The band around 251 nm is due to an *n*‐*n*^∗^ transition in the carbonyl chromophore while the band at 312 nm can be assigned to MLCT transition from the hydroxy group to Cu(II) ions. The positive Cotton effect around 750 nm may be attributed to the helical chain which is consistent with the right-handed supramolecular framework (Figure 2(c)) [30, 31]. Time-dependent CD measurement shows that the absolute values of the ellipticity around 251 nm, 312 nm, and 750 nm of both 1 and 2 were greatly reduced after irradiation (Figure 2(c)–(d)).

### 2.5. Mechanism Study of Photoresponsive Behavior

In order to study the mechanism of the photochromic process of the chiral products, IR and PXRD analysis was carried out and the single-crystal X-ray diffraction data of irradiated samples were also collected. For compound 1, no obvious variation was found in the PXRD patterns (Figure S10) and IR spectra (Figure S11) during the process of photochromism. Adding that into consideration with a T type photochromic behavior, it can be confirmed that molecular structures of bulk samples of 1 remain intact after irradiation and the photochromic behavior originates from photoinduced electron transfer rather than photolysis or rearrangement reactions. SCXRD analysis also reveals that crystal structures of 1 and 1P have the same coordination modes with a subtle difference in cell parameters, bond lengths, and bond angles. However, almost all C-O bonds of the malate ligand shrink trivially (Figure S1) while the bond lengths of C13-C16 bonds increase from 1.484(6) to 1.495(8) Å, and the dihedral angle of the two pyridine rings of the bpy ligands increases from 12.4(6) to 13.5(9)° after irradiation (Figure 3(a)). Such changes and trends in the structure are also characteristic of electron transfer photochromic species although the shifts are too small to be sufficient evidence [32, 33]. In addition, as a kind of electron-rich component, oxygens of carboxylate can serve as electron donors while coordinated bpy ligands can accept electrons under the electron-withdrawing effect of Cu(II). The potential donor-acceptor pair also increases the possibility of photoinduced electron transfer (PET) during the irradiation process.

In most PET systems, radicals are generated concomitantly during the irradiation process. To detect the photogenerated radicals, EPR measurements were conducted. Solid-state EPR spectra of compound 1 were collected with powder samples in quartz tubes with a 9.839 GHz magnetic field at room temperature. The spectral data of irradiated compound 1P were collected with the same samples upon rolling and UV-vis irradiation for half an hour. As shown in Figure 3(b), the EPR spectra of 1 show a typical axial symmetry signal of Cu(II) ions, with two signal peaks located at *g*_‖_ = 2.234 and *g*_*⊥*_ = 2.103, respectively. The geometric parameter *G* is 2.30 < 4, calculated with the equation *G* = (*g*_‖_ − 2.0023)/(*g*_*⊥*_ − 2.0023) indicating a strong coordination anisotropy of Cu(II) ions. Unfortunately, radical signals were not found in the spectra of 1P, a weak signal was detected, and there is only a tiny difference in the *g*_‖_ parts of the Cu(II) signal between the EPR spectrum of 1P and 1. A rational explanation is that the strong signals of Cu(II) ions overlapped with the radicals while the conversion rate of the latter is far from 100%. Meanwhile, the occurrence of electron transfer from ligands to Cu(II) ions can also be excluded.

To further verify our suggestion, low-temperature EPR measurements were conducted with a 9.834 GHz magnetic field. Before irradiation, the strong signals of Cu(II) ions corresponding to *g*_‖_ = 2.234 and *g*_*⊥*_ = 2.110 were sharper than those at room temperature. After irradiation, the intensity of the EPR signal drastically weakened to only 0.75% of that of unirradiated samples (Figure 3(c)). Combined with RT data, the reduced signals are in accord with the magnetic coupling between Cu(II) ions and radicals. At a low temperature, a stronger coupling results in a huge decrease in the intensity of the EPR signals. Similar results were also observed in some Mn(II) complexes with strong magnetic interactions between metal ions and radicals [34].

**Figure 4:**
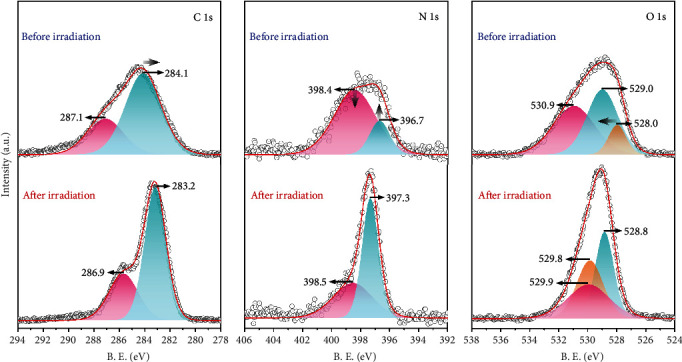
XPS core-level spectra of 1 before and after irradiation (1P). The dots and red lines depict the experimental data and sum of simulated resolved peaks, respectively.

XPS measurements were carried out to find more evidence to support our assertion. The EPR study suggests that copper atoms remain divalent after the coloration, while organic components usually participate in the PET process. Therefore, the XPS spectra of irradiated samples are unsuitable to reference to the C 1s neutral carbon peak at 284.6 eV, while 1P referenced to Cu 2p_3/2_ of unirradiated samples and background was deducted (Figure S12). In addition, the binding energy of auger peak of Cu LLM was also found around 570 eV, and no active signal was observed at 340 eV before and after irradiation, which implies that the valence of Cu(II) ions is unchanged upon irradiation. As shown in Figure 4, the binding energy of C 1s and N 1s shows a general decrease while that of O 1s shows an increase after irradiation indicating PET processes occurred. For compound 1, two fitting peaks of the C 1s at 287.1 eV and 284.1 eV are attributable to C atoms of malate ions and C atoms of bpy moieties, respectively. The intensity ratio of the two peaks is 0.44 (*I*_287.1_/*I*_284.1_), well-correlated with the theoretical value in the compound (0.4). After irradiation, the low energy fitting peak of 1P shifted to 283.2 eV, suggesting pyridyl C atoms are part of the electron acceptors. The N 1s spectrum of 1 can be fitted to two peaks at 398.4 eV and 396.7 eV with an intensity ratio of 3.89 (*I*_398.4_/*I*_396.7_) while that of 1P shows two fitting peaks at 398.5 eV and 397.3 eV with an intensity of 0.69 (*I*_398.5_/*I*_397.3_). The significant difference of the low energy band shifts and intensity ratio may result from a majority of peaks at 398.4 eV shifted to low energy and overlapping with the weak peak originally at 397.3 eV. Usually, the spectra of irradiated samples in PET systems result from the behavior of a mixture of several states (ground states, excited states, and charge-separated states); therefore, it is difficult to assign the split peak. However, N atoms are only from bpy moieties in compound 1, and the overall decrease of the binding energy of N 1s band after irradiation indicates that pyridyl N atoms are also components of electron acceptors during the PET process. The O 1s spectrum of 1 is complicated due to the existence of oxygen atoms in multiple chemical environments. However, the overall shifts of O 1s peaks to high binding energy after irradiation suggest that part of O components is acting as electron donors in the PET process.

**Figure 5:**
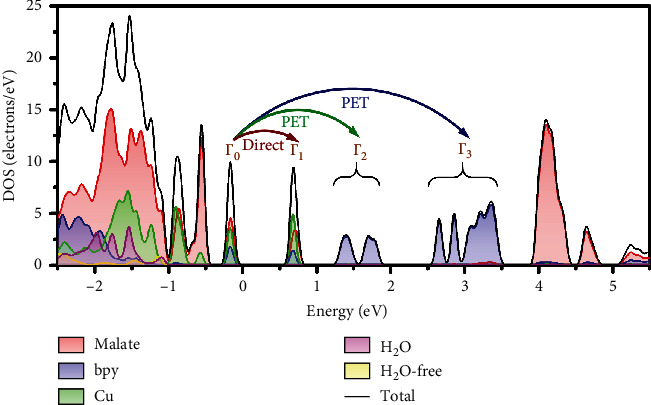
Total and partial density of states (PDOS) of 1.

To further confirm the donors and acceptors, the total and partial density of states (DOS) (Figure 5) and band structures (BS) (Figure S13) of 1 were calculated. There is little dispersion in the lattice due to the large cell volume of 1. The calculated band structure is in overall agreement with that of the DOS. Both the valence band maximum (VBM) of 1 and the conduction band minimum (CBM) are mainly composed of orbitals of copper ions and the oxygen atoms of malate ligands. The band gap between VBM and CBM is only 0.56 eV (4516 cm^−1^, 2214 nm), corresponding to Γ_0_ to Γ_1_ (4677 cm^−1^, 2138 nm) in the band structure. Such a small band gap facilitates the vertical energy transition of electrons of copper and malate oxygen, respectively, from the ground state to the lowest excited state. However, no obvious shifts and new absorption bands were observed on the time-dependent IR and UV-vis data (Figure S14). This might be due to the short lifetime of the photoexcited states with Cu(II) ions only acting as catalysts in this process [35, 36]. The second conduction band (CB) (Γ_0_ to Γ_2_, 991 nm) and the third CB (Γ_0_ to Γ_3_, 481 nm) transitions are almost contributed by bpy ligands, especially the pyridyl carbons. The band gaps between the VBM and the second CB and the third CB are 1.23 eV and 2.53 eV, which correspond to the energies of light with wavelengths of 1010 nm and 491 nm, respectively. The latter is similar to the variation of electronic absorption spectra. The absorption band of Γ_0_ to Γ_4_ is 3.95 eV (314 nm) and was close to the absorption band observed in the UV-vis spectrum. In consideration of the result of XPS and EPR analysis, the direct redox reaction of Cu(II) ions can be excluded upon the photochromic process. Hence, we suggest that the photochromism of 1 and 2 was derived from a PET process. Upon irradiation, electrons transferred from oxygen atoms of malate ligands to the pyridyl carbon atoms (major) and pyridyl nitrogen atoms (minor). Hence, the increased C-C bonds and dihedral angles of 1 after irradiation can be explained. The bpy ligands received electrons from malate ions during the PET process resulting in an increased repulsion of two pyridyl rings (Figure 3(a)). In the crystal structure, the short contacts between these atoms can provide suitable pathways for electron transfer, which is consistent with the XPS results (Figure S15). Moreover, it is worth noting that in the wavelength study, compound 1 is most sensitive to light *λ* = 350 nm (Figure S5) which is near to the energy gap between Γ_0_ and Γ_4_ in the DOS. In consideration of the constitution of Γ_0_ and Γ_4_, such a result demonstrates that the valence electron in donor atoms' HOMO (D-A) was firstly irradiated to excited states (D^∗^-A) and then transitioned to and stabilized in acceptor atoms' LUMO (D^+^-A^−^). This process is in accord with the PET process in other photochromic compounds [9].

### 2.6. Study of Photodynamics

In the PET system, the reaction progress monitoring should be focused on the concentration or the production rate of photogenerated radicals which can be approximately calculated by the *χ*_*M*_*T* − *T* curves. The contribution to the *χ*_*M*_*T* value of a free radical is c.a. 0.375 cm^3^ mol^−1^ K at room temperature. Thus, the maximum number of photogenerated radicals in a molecular *Q* = (*χ*_*M*_*T*(1*P*) − *χ*_*M*_*T*(1))/(*χ*_*M*_*T*(radicals)) is 0.568 at room temperature. In the combination of changes of values at *λ* = 448 nm, UV-vis absorption spectra, the kinetic curves of electron transfer ratio can be drawn (Figure S16). By fitting the plots in time-dependent UV-vis spectra curves with first-order reaction kinetics equations, the reaction rate constant *k*_abs_ of PET reactions is 7.526 × 10^−4^ s^−1^.

The lifetime of irradiated states was also investigated by a temperature-dependent study. To ensure a complete colorization process and avoid the interference of the photothermal effect, the fresh crystal samples were collected and grind to powders, then irradiated upon UV light with continuous stirring for 5 hours. As shown in Figure S17, irradiated green samples can generally return to blue upon heating. The needed annealing time distributes from 30 to 160 min when the temperature ranges from 90 to 50°C. The annealed samples can change their color to green again after irradiated by the UV light for an hour. The metastability between the two states could provide some application potential in sensing and switching.

## 3. Discussion

Compound 1 can generate radicals and lead to CS during the PET process. The difference in electronic structures between the GS and CS will certainly produce photochromic behavior and be reflected in the UV-vis absorption spectra. As new spin carriers, photogenerated radicals also result in the increase of the magnetic susceptibilities after irradiation. As mentioned above, it was confirmed that -COO^−^ and -OH groups as donors were involved in PET upon irradiation. Since the Cotton effects of chiral materials are greatly affected by their absorption/asymmetrical transition, the decrease of intensity in these two bands is expectable [37]. Along with the radicals generated in the CS states, in turn, the original dipole transitions with -OH and -COO^−^ groups were reduced; smaller contributions in the Cotton effects from both compounds result in a decreased intensity in the CD spectra upon irradiation.

Although there are several examples of successful construction of photochromic compounds with nonphotochromic reagents, most other bipyridine-based complexes that meet the requirements of metalloviologen in structures are all nonphotochromic [23–26]. It is still challenging to explore new metalloviologen compounds with photochromism. The building units of both enantiomeric compounds reported here are also nonphotochromic. Understanding the underlying mechanisms of these examples will provide insights about the targeted synthetic strategies. The design of stimulus-responsive chemical species with chiroptical variation at the molecular level is critical for chiral switching. In most other photoresponsive chiral MOFs, the chiral symmetry and pore structures are used for the study of chiral separation and asymmetric catalysis; however, the switchable chiroptical properties still lacks of study [38, 39]. Though similar chiroptical behaviors were previously found in some small molecular systems, such as diarylethene (DTE), azo-compounds, and 2+2 cycloaddition systems [40–46], these systems do not involve a PET process due to the difficulty in ensuring the contribution of chiral chromophores, especially in the solid state. More importantly, the solid-state molecular switch materials with differing electron states could show better fatigue resistance than those with distinct molecular structures due to a lower mecho-chemical effect [47, 48]. In this regard, both 1 and 2 represent a new system for the design of chiral optical molecular switches based on MOFs. A synergistic optical response of photochromism, photomagnetism, and chiroptical variation due to PET makes compounds 1 and 2 good candidates as multifunctional smart materials. In addition, the introduction as well as the participation of chiral units in the PET process will provide other potential functionality related to polar groups such as nonliner optics [49].

## 4. Conclusion

In summary, by the introduction of chirality into a D-M-A system and rational selection of each component, we have successfully constructed two enantiomeric MOFs showing photochromic, photomagnetic, and chiroptical properties at room temperature concurrently. All three photoresponsive behaviors were triggered by a PET process. As expected, the selected donor ligands and acceptor ligands coordinated with copper centers and occupied all coordination sites of copper ions with coordination geometry in the equatorial plane. The Jhan-Teller effect of Cu(II) cations produces short contacts between donor and acceptor components in the structures. Upon irradiation, photoinduced electron transfer from oxygen atoms of chiral carboxylic ligands to N-heterocycles was observed, and radicals were delocalized in the aromatic ring. The magnetic exchange between radicals and metal ions results in reduced signals in the EPR spectra and enhanced magnetic susceptibility. The dipole transitions of chiral chromophores were also influenced so that the Cotton effects of chiral compound were weakened upon irradiation. This work opens up a new pathway for designing chiral stimuli-responsive materials via the D-M-A strategy. The combination of photomagnetic and chiroptical properties of MOFs 1-2 at room temperature promotes potential applications in the fields of photocontrolled dual-functional switches and information storage.

## 5. Materials and Methods

### 5.1. Synthesis

*Preparation of compound 1*. Zavakhina's group previously synthesized compound Cu_2_(L-mal)_2_(bpy)_2_(H_2_O)·2.5H_2_O by heating the mixture of malic acid, bpy, and basic cupric carbonate [28]. However, according to their report, the main product is amorphous precipitate with a very few crystals. We here use a diffusion method and successfully get pure bulk crystalline products. A mixture of Cu(OH)_2_·CO_3_ (2.5 mmol, 553 mg), L-malic acid (12 mmol, 1609 mg), and aqueous solution of DMSO (v/v 1 : 1, 50 mL) was added into a 100 mL flask and refluxed for half hour, then cooled to room temperature and filtrated; cyan solution of copper malate was obtained and divided into 10 pieces. Each piece was added in one arm of a 20 mL H-shaped pipe while an aqueous solution of DMSO (v/v 1 : 1, 5 mL) containing 0.5 mmol 4,4′-bipyridine (0.078 g) was added in another arm; then, 6 mL aqueous solution of EtOH (v/v 1 : 1) was carefully added in both sides as a buffer and sealed with parafilm (Scheme S1). After slow diffusion for two months, blue needle crystals of 1 were obtained with a yield of 55% (based on Cu^2+^). The phase purity of bulk crystalline samples was checked by powder X-ray diffraction (PXRD) (Figure S10) and elemental analyses. Anal. calcd for C_28_H_32_Cu_2_N_4_O_14_ (1) (%): C 43.36, H 4.16, N 7.22; found (%): C 42.66, H 3.95, N 7.31.

*Preparation of compound 2*. Compound 2 is the enantiomer of 1 and was synthesized in the same manner by using D-malic acid instead of L-malic acid. Anal. calcd for C_28_H_32_Cu_2_N_4_O_14_ (2) (%): C 43.36, H4.16, N 7.22; found (%): C 43.40, H 4.68, N 7.30.

*Preparation of compound 3*. Compound 3 can be obtained in the same manner as compound 1 using DL-malic acid instead of L-malic acid. However, bulk pure products can hardly be isolated due to a single-crystal single-crystal (SCSC) transition-like process existed in synthesis. After diffusion for a day, bulk light blue block crystals with chemical components of Cu(DL-Hmal)_2_(H_2_O)_2_ were crystallized on the bottom of the tube (Figure S18) [50, 51]. Then, as time goes on, a mixture of blue block crystals of 3 grew on the surface of light blue block crystals (Figure S2). Suitable single crystal of 3 can be carefully taken off from a solid mixture. The same result can also be observed by immersing crystals of Cu(DL-Hmal)_2_(H_2_O)_2_ in the EtOH solution of bpy. Anal. calcd for C_14_H_12_CuN_2_O_5_ (3) (%): C 47.8, H 3.44, N 7.96; found (%): C 42.14, H 4.30, N 6.76.

### 5.2. Characterization

The C, H, N elemental analyses were measured using an Elementar Vario EL Cube analyzer. Powder X-ray diffraction (PXRD) patterns were collected at room temperature on a Rigaku D/max-2500 diffractometer using Cu-K*α* radiation (*λ* = 1.54184 Å). The simulated PXRD patterns were derived from the free Mercury software. UV–vis absorption spectra were recorded at room temperature on a Persee TU1901 UV-vis spectrophotometer with an integrating sphere attachment and BaSO_4_ pellets as a background in the regions 300-800 nm regions and on a SHIMADZU UV-3600 UV-vis spectrophotometer in the regions of 1800-2500 nm. The FT-IR spectra were recorded using powder samples on a Bruker TENSOR II FT-IR spectrophotometer in the 4000–400 cm^−1^ regions. Time-dependent FT-IR spectra were measured on a Bruker VERTEX 70 FT-IR spectrophotometer in the region of 6000–3000 cm^−1^ with KCl pellets. The solid-state circular dichroism (CD) spectra were measured using a Jasco J-715 circular dichroism spectrometer with KCl pellets (2 mg sample in 50 mg KCl) with a bandwidth of 10 nm at room temperature. The electron paramagnetic resonance (EPR) spectra were recorded on a Bruker EMX-plus spectrometer with the same power and parameters for samples before and after irradiation. X-ray photoelectron spectroscopy (XPS) studies were performed with a Thermo Scientific ESCALAB 250Xi X-ray photoelectron spectrometer using Al K*α* radiation (*λ* = 8.357 Å). A 300 W xenon lamp (Perfect Light PLS-SXE 300C) system equipped with filter was used to illuminate samples for achieving various spectra, and the distances between these samples and the Xe lamp were *c.a.* 30 cm to reduce the photothermal effect. Three types of filters including UV-cut (>360 nm), UV-pass (<360 nm), and IR-cut (<850 nm) band filter can be equipped with Xenon lamp to get UV (146 mw/cm^2^), visible (712 mw/cm^2^), and UV-vis (935 mw/cm^2^) light source, respectively. The magnetic data were recorded on a Quantum Design SQUID MPMS-5 magnetometer. Diamagnetic corrections were made with Pascal's constants for all the constituent atoms. The magnetic data, PXRD, SCRD, IR, XPS, and EPR data of irradiated samples were collected immediately right after crystalline samples were irradiated by using a xenon lamp.

### 5.3. Computation Method

Within first-principle calculations, the Projector Augmented-Wave (PAW) method [52] was used to describe the ion-electron interaction as implemented in the Vienna *ab* initio simulation package (VASP) [53, 54]. The Perdew-Burke-Ernzerhof (PBE) [55] exchange-correlation functional and a 520 eV energy cutoff plane-wave basis set were adopted to perform the calculations. The model is a hexagonal cell with lattice parameters of *a* = *b* = 11.20 Å and *c* = 43.50 Å; Brillouin zone was sampled by a 3 × 3 × 1Γ-centered mesh. During geometry optimization, all the atoms were relaxed till the force on each atom is less than 0.05 eV/Å in each direction, and the energy convergence criterion was set to 10^−5^ eV in all calculations.

### 5.4. X-ray Crystallography

Single crystal X-ray diffraction data were collected on a computer-controlled Rigaku XtalAB PRO MM007 DW diffractometer equipped with graphite-monochromated Cu-K*α* radiation with a radiation wavelength of 1.54184 Å. Lorentz polarization and absorption corrections were applied. The crystal structure data were solved by direct methods using the *SHELXS* program of the *SHELXTL* package and refined by full-matrix least-squares methods with *SHELXL* [56]. The nonhydrogen atoms were located in successive difference Fourier syntheses and refined with anisotropic thermal parameters on *F*^2^. Hydrogen atoms were generated theoretically at specific atoms and refined isotropically with fixed thermal factors. The squeeze method was conducted with the *Platon* program for compound 3 [57]. The X-ray crystallographic coordinates for structures reported in this article have been deposited at the Cambridge Crystallographic Data Centre (CCDC), under deposition nos. CCDC: 1969123 (1), 1969124 (1P), 1969125 (2), and 1969126 (3).

## Data Availability

All data needed in the paper are present in the paper and in the Supplementary section. Additional data which are related to this paper may be requested from the authors.

## Supplementary Materials

Supplementary Materials Description of the detailed crystal structure. Table S1. Crystal data and structure refinement parameters. Table S2. Selected bond lengths (Å) and angles (°) for complex 1 (a), 1P (b), 2 (c), and 3 (d). Figure S1. Structure of 1. (a) Coordination environments of the metal ion in 1 (H atoms are omitted). Symmetry code: #1 x-1, y, z; #2 x+1, y, z; #3 x-y, x-1, z+1/6; #4 y+1, -x+y+1, z-1/6. (b) Coordination modes of malate ions. (c) Projection of 1 on the *ab* plane and 1D-chiral channels at the *c* axis. (d) (5-c)(4-c)(3-c) topological framework of 1. (e) Structure variation upon irradiation. Figure S2. The symbiosis of Cu(DL-Hmal)_2_(H_2_O)_2_ (light blue) and 3 (blue). Figure S3. Structure of 3. H atoms and disordering of ligands are omitted for clarity. Symmetry code: #1 1-x, 1-y, 1-z; #2 1-x, 2-y, 1-z; #3 x+1, y, z. (a) Coordination environments of the metal ion in 3. (b) 2D layer of [Cu(DL-mal)]_n_. (c) View of 3 on the *bc* plane. (d) The topology of 3. Nodes: 6-c copper (red), 4-c copper (blue), 3-c malate (black). (e) Disordering of malate and bpy ligands in compound 3. Figure S4. UV-vis absorption spectra of 1 upon visible light irradiation. Figure S5. The color change of 1 upon irradiation for 15 min with light at variable wavelength. Figure S6. Curie-Weiss Fitting of magnetic data for 1 (a) and 1P (b). Figure S7. Plots of *χ*_M_ and *χ*_MT_ versus T for 1. The solid line is the best fit to the experimental data by using the spin Hamiltonian operator. Figure S8. Magnetization (M) versus field (H) for 1 and 1P at 2 K. Figure S9. Solid-state CD spectra of 1, 1P, 2, and 2P. Figure S10. PXRD of 1 and 2 before and after irradiation and annealed samples (1A). Figure S11. FT-IR spectra of 1, 1P, 2, and 2P. Figure S12. Cu 2p (a), Cu (II) LLM(b), and Cu(I) LLM (c) partial XPS spectra of 1 and 1P. Figure S13. Band structure of 1. Figure S14. Time-dependent FT-IR spectrum of 1 with KCl Pellets (1 : 100) (a) and local UV-vis spectrum (b) of 1 upon UV-vis light irradiation. Figure S15. Possible electron transfer passway of 1 during the photochromic process. Figure S16. Kinetics of photoinduced electron transfer of 1. Left: Number of photogenerated radicals (red) for a Cu_2_(L-mal)_2_(bpy)_2_(H_2_O)·3H_2_O upon irradiation. Right: First-order kinetic plot (blue) for change in the absorbance at *λ* = 448 nm, where A_0_, A_t_, and A_∞_ are the absorbance values at time zero, time *t*, and infinite time of the reaction, respectively. Figure S17. The color change of 1 upon irradiation and heating at variable temperature. Scheme S1. Schematic diagram of the diffusion method for preparations of 1-3. Figure S18. Structure of Cu(Hmal)_2_(H_2_O)_2_.
