# Supplementary material for: Concomitant Photoresponsive Chiroptics and Magnetism in Metal-Organic Frameworks at Room Temperature
Source: Research (Wash D C). 2021 Feb 10;2021:5490482. doi: 10.34133/2021/5490482 (PMC7894082; doi:10.34133/2021/5490482)
Supplement: Supplementary Materials — Description of the detailed crystal structure. Table S1. Crystal data and structure refinement parameters. Table S2. Selected bond lengths (Å) and angles (°) for complex 1 (a), 1P (b), 2 (c), and 3 (d). Figure S1. Structure of 1. (a) Coordination environments of the metal ion in 1 (H atoms are omitted). Symmetry code: #1 x-1, y, z; #2 x+1, y, z; #3 x-y, x-1, z+1/6; #4 y+1, -x+y+1, z-1/6. (b) Coordination modes of malate ions. (c) Projection of 1 on the ab plane and 1D-chiral channels at the c axis. (d) (5-c)(4-c)(3-c) topological framework of 1. (e) Structure variation upon irradiation. Figure S2. The symbiosis of Cu(DL-Hmal)2(H2O)2 (light blue) and 3 (blue). Figure S3. Structure of 3. H atoms and disordering of ligands are omitted for clarity. Symmetry code: #1 1-x, 1-y, 1-z; #2 1-x, 2-y, 1-z; #3 x+1, y, z. (a) Coordination environments of the metal ion in 3. (b) 2D layer of [Cu(DL-mal)]n. (c) View of 3 on the bc plane. (d) The topology of 3. Nodes: 6-c copper (red), 4-c copper (blue), 3-c malate (black). (e) Disordering of malate and bpy ligands in compound 3. Figure S4. UV-vis absorption spectra of 1 upon visible light irradiation. Figure S5. The color change of 1 upon irradiation for 15 min with light at variable wavelength. Figure S6. Curie-Weiss Fitting of magnetic data for 1 (a) and 1P (b). Figure S7. Plots of χM and χMT versus T for 1. The solid line is the best fit to the experimental data by using the spin Hamiltonian operator. Figure S8. Magnetization (M) versus field (H) for 1 and 1P at 2 K. Figure S9. Solid-state CD spectra of 1, 1P, 2, and 2P. Figure S10. PXRD of 1 and 2 before and after irradiation and annealed samples (1A). Figure S11. FT-IR spectra of 1, 1P, 2, and 2P. Figure S12. Cu 2p (a), Cu (II) LLM(b), and Cu(I) LLM (c) partial XPS spectra of 1 and 1P. Figure S13. Band structure of 1. Figure S14. Time-dependent FT-IR spectrum of 1 with KCl Pellets (1 : 100) (a) and local UV-vis spectrum (b) of 1 upon UV-vis light irradiation. Figure S15. Possib [file 5490482.f1.docx]

**Supporting Information**

**Concomitant Photo-responsive Chiroptics and Magnetism in Metal-Organic Frameworks at Room Temperature**

Bin Xia^1^, Qian Gao^3^, Zhen-Peng Hu^3^, Qing-Lun Wang^1*^, Xue-Wei Cao^3^, Wei Li^2^, You Song^4^ & Xian-He Bu^1,2*^

^1^College of Chemistry, State Key Lab of Elemento-Organic Chemistry, Nankai University, Tianjin 300071, China.

^2^School of Materials Science and Engineering, Tianjin Key Lab of Metal and Molecule-Based Material Chemistry, Nankai University, Tianjin 300350, China.

^3^School of Physics, Nankai University, Tianjin 300071, China.

^4^State Key Lab of Coordination Chemistry, School of Chemistry and Chemical Engineering, Nanjing University, Nanjing 210023, China.

*Email: [wangql@nankai.edu.cn](mailto:wangql@nankai.edu.cn) (Q.-L. Wang); [buxh@nankai.edu.cn](mailto:buxh@nankai.edu.cn) (X.-H. Bu).

**Detailed crystal structure of compound 3**

Crystal structure of **3**. Compound **3** crystallizes in the triclinic space group *P*‾1 (No. 2). The asymmetric unit of **3** consists two half copper(II) cations, one malate ions and two half bpy ligands. Due to the existence of racemic ligands and the centrosymmetric *P*‾1 group imposing *C_i_* point symmetry on the malate ion, seriously orientational disordering of malate ions and bpy ligands were observed in the crystal structure. Each Cu1 atom is ligated by two O5 atoms from different malate ions and two N5 atoms from two different bpy molecules and lies in a square planar [CuN_2_O_2_] coordination geometry. Each Cu2 atom is six-coordinated with four carboxylic oxygens (two O2, two O6) and two bipyridyl nitrogens (two N6) in an elongated octahedral [CuN_2_O_4_] coordination geometries. The malate ions adopt a monodentate-bidentate mode and serves as three connected nodes. Two neighboring Cu2 atoms are connected by two malate ligands end-to-end to consist a small ring structure while a large ring structure were connected with two neighboring Cu2 atoms and two neighboring Cu1 atoms by two malate ions. Each small ring is fused to four big rings to give an edge-shared terraced [Cu(DL-mal)]n network with **cpa** topology on the ab plane. The 2D network was further connected with bpy ligands in c axis to construct a 3D framework. Since the disorder in structure makes two oppsite connected orientations of malate ions, Cu1 and Cu2 atoms serve as 4-c and 6-c centers in turn between the adjacent [Cu(DL-mal)]_n_ network. With addition of 3-c nodes of malates, the whole framework presents an unreported topology of point symbol {4·6^2^}^2^{4^2^·6^8^·8^3^·10^2^}{6^4^·8^2^} (td10 = 1170). The vertexes and construction units of the new topology were similar with **tpz**, **sqc130**, **sqc131** and **sqc180** topology but different in connecting modes[1-5].

**References**

[1] T. G. Akhmetshina, V. A. Blatov, D. M. Proserpio and A. P. Shevchenko, "Topology of Intermetallic Structures: From Statistics to Rational Design," - *Accounts of Chemical Research* , vol. 51, no. 1, pp. 21-30, 2018.

[2] S. J. Ramsden, V. Robins and S. T. Hyde, "Three-dimensional Euclidean nets from two-dimensional hyperbolic tilings: kaleidoscopic examples," *Acta Crystallographica Section A Foundations of Crystallography*, vol. 65, no. 2, pp. 81-108, 2009.

[3] K. Nath, A. Husain and P. Dastidar, "Metallogels and Silver Nanoparticles Generated from a Series of Transition Metal-Based Coordination Polymers Derived from a New Bis-pyridyl-bis-amide Ligand and Various Carboxylates," *Crystal Growth & Design*, vol. 15, no. 9, pp. 4635-4645, 2015.

[4] S. Shin, S. Jeong, D. Kim and M. S. Lah, "Reversible Single-Crystal-to-Single-Crystal Transformations of Metal–Organic Frameworks that Accompany Two-Dimensional Framework Reorganizations," *Crystal Growth & Design*, vol. 17, no. 4, pp. 2228-2237, 2017.

[5] A. K. Gupta, K. Tomar and P. K. Bharadwaj, "Cd(ii) coordination polymers constructed with a flexible carboxylate linker and pyridyl co-linkers: variation in the network topologies and photoluminescence properties," *CrystEngComm*, vol. 19, no. 16, pp. 2253-2263, 2017.

**Tables**

**Table S1** Crystal data and structure refinement parameters

| Compound | **1** | **1P** | **2** | **3** |
| --- | --- | --- | --- | --- |
| Empirical formula | C_28_H_32_Cu_2_N_4_O_14_ | C_28_H_32_Cu_2_N_4_O_14_ | C_28_H_32_Cu_2_N_4_O_14_ | C_14_H_12_CuN_2_O_5_ |
| Formula weight | 775.65 | 775.65 | 775.65 | 351.80 |
| Temperature | 100 K | 100 K | 120 K | 300 K |
| Crystal system | Hexagonal | Hexagonal | Hexagonal | Triclinic |
| Space group | *P6_1_* | *P6_1_* | *P6_5_* | *P‾1* |
| *a*, Å | 11.12880(10) | 11.12210(10) | 11.12590(10) | 8.1479(6) |
| *b*, Å | 11.12880(10) | 11.12210(10) | 11.12590(10) | 9.6867(7) |
| *c*, Å | 41.8505(6) | 41.9663(6) | 41.6907(5) | 11.1166(10) |
| *α*, deg | 90 | 90 | 90 | 72.125(7) |
| *β*, deg | 90 | 90 | 90 | 76.029(7) |
| *γ*, deg | 120 | 120 | 120 | 78.942(6) |
| *V*, Å^3^ | 4488.77(10) | 4495.78(10) | 4469.31(10) | 803.73(12) |
| Z | 6 | 6 | 6 | 2 |
| *ρ*_calcd_, g/cm^3^ | 1.722 | 1.719 | 1.729 | 1.454 |
| *μ*,mm^−1^ | 2.463 | 2.459 | 2.474 | 2.141 |
| F(000) | 2388 | 2388 | 2388 | 358 |
| *θ* range collected, deg | 4.588-73.423 | 4.591 - 73.602 | 4.589 - 73.397 | 4.261 - 73.644 |
| Data/unique | 16171/5700 | 16188/5489 | 14987/5739 | 7057/3096 |
| R_int_ | 0.0288 | 0.0455 | 0.0142 | 0.0366 |
| Data/restraints/parameters | 5700/25/435 | 5489/25/435 | 5739/1/435 | 3096/0/337 |
| GOF | 1.044 | 1.029 | 1.085 | 0.912 |
| Completeness to *θ*= 67.679 | 100.0 % | 100.0 % | 99.9% | 98.9 % |
| *R*_1_/w*R*_2_ [*I*> 2*σ*(*I*)] | 0.0328/0.0840 | 0.0512/0.1373 | 0.0239/0.0642 | 0.0760/0.2140 |
| *R*_1_/w*R*_2_ (all data) | 0.0331/0.0842 | 0.0518/0.1378 | 0.0240/0.0643 | 0.0958/0.2335 |
| Flack | 0.004(12) | -0.03(3) | -0.012(6) | - |

**Table S2a**. Selected bond lengths (Å) and angles (°) for complex **1**

| Bond lengths | | | |
| --- | --- | --- | --- |
| Cu(1)-O(9) | 1.958(3) | Cu(1)-O(6) | 1.969(3) |
| Cu(1)-N(1) | 2.025(4) | Cu(1)-N(2) | 2.028(3) |
| Cu(1)-O(1) | 2.245(3) | Cu(2)-O(4) | 1.964(3) |
| Cu(2)-O(2) | 1.964(3) | Cu(2)-N(4) | 2.016(3) |
| Cu(2)-N(3) | 2.022(4) | Cu(2)-O(11) | 2.330(4) |
| Bond angles | | | |
| O(9)-Cu(1)-O(6) | 177.67(13) | O(9)-Cu(1)-N(1) | 89.19(14) |
| O(6)-Cu(1)-N(1) | 92.03(14) | O(9)-Cu(1)-N(2) | 91.19(14) |
| O(6)-Cu(1)-N(2) | 87.48(14) | N(1)-Cu(1)-N(2) | 176.94(15) |
| O(9)-Cu(1)-O(1) | 90.17(13) | O(6)-Cu(1)-O(1) | 91.81(13) |
| N(1)-Cu(1)-O(1) | 89.90(13) | N(2)-Cu(1)-O(1) | 93.13(13) |
| O(4)-Cu(2)-O(2) | 174.87(13) | O(4)-Cu(2)-N(4) | 92.68(14) |
| O(2)-Cu(2)-N(4) | 92.25(13) | O(4)-Cu(2)-N(3) | 86.74(14) |
| O(2)-Cu(2)-N(3) | 88.65(14) | N(4)-Cu(2)-N(3) | 170.44(16) |
| O(4)-Cu(2)-O(11) | 91.57(14) | O(2)-Cu(2)-O(11) | 87.11(13) |
| N(4)-Cu(2)-O(11) | 88.34(14) | N(3)-Cu(2)-O(11) | 101.21(15) |
| C(5)-N(1)-Cu(1) | 117.2(3) | C(1)-N(1)-Cu(1) | 125.0(3) |
| C(8)-N(2)-Cu(1) | 120.1(3) | C(9)-N(2)-Cu(1) | 121.9(3) |
| C(11)-N(3)-Cu(2) | 122.1(3) | C(15)-N(3)-Cu(2) | 120.0(3) |
| C(19)-N(4)-Cu(2) | 119.0(3) | C(18)#2-N(4)-Cu(2) | 123.4(3) |
| C(21)-O(2)-Cu(2) | 114.6(3) | C(21)-O(1)-Cu(1) | 173.9(3) |
| C(25)-O(6)-Cu(1) | 110.8(3) | C(24)-O(4)-Cu(2) | 129.3(3) |
| C(28)-O(9)-Cu(1) | 120.8(3) |  |  |
| Symmetry transformations used to generate equivalent atoms: #1 x-1,y,z;#2 x+1,y,z; #3 x-y,x-1,z+1/6; #4 y+1,-x+y+1,z-1/6. | | | |

**Table S2b**. Selected bond lengths (Å) and angles (°) for complex **1P**

| Bond lengths | | | |
| --- | --- | --- | --- |
| Cu(1)-O(6) | 1.965(4) | Cu(1)-O(9) | 1.964(5) |
| Cu(1)-N(2) | 2.027(5) | Cu(1)-N(1) | 2.030(5) |
| Cu(1)-O(1) | 2.252(5) | Cu(2)-O(4) | 1.964(5) |
| Cu(2)-O(2) | 1.976(5) | Cu(2)-N(4) | 2.019(5) |
| Cu(2)-N(3) | 2.025(5) | Cu(2)-O(11) | 2.339(6) |
| Bond angles | | | |
| O(9)-Cu(1)-O(6) | 178.15(18) | O(9)-Cu(1)-N(1) | 89.1(2) |
| O(6)-Cu(1)-N(1) | 92.1(2) | O(9)-Cu(1)-N(2) | 91.1(2) |
| O(6)-Cu(1)-N(2) | 87.6(2) | N(1)-Cu(1)-N(2) | 176.8(2) |
| O(9)-Cu(1)-O(1) | 90.14(19) | O(6)-Cu(1)-O(1) | 91.31(19) |
| N(1)-Cu(1)-O(1) | 90.0(2) | N(2)-Cu(1)-O(1) | 93.22(19) |
| O(4)-Cu(2)-O(2) | 174.7(2) | O(4)-Cu(2)-N(4) | 92.5(2) |
| O(2)-Cu(2)-N(4) | 92.5(2) | O(4)-Cu(2)-N(3) | 86.8(2) |
| O(2)-Cu(2)-N(3) | 88.6(2) | N(4)-Cu(2)-N(3) | 170.3(2) |
| O(4)-Cu(2)-O(11) | 91.4(2) | O(2)-Cu(2)-O(11) | 87.1(2) |
| N(4)-Cu(2)-O(11) | 88.5(2) | N(3)-Cu(2)-O(11) | 101.2(2) |
| C(5)-N(1)-Cu(1) | 117.3(4) | C(1)-N(1)-Cu(1) | 124.6(4) |
| C(8)-N(2)-Cu(1) | 120.2(4) | C(9)-N(2)-Cu(1) | 121.4(4) |
| C(11)-N(3)-Cu(2) | 122.0(4) | C(15)-N(3)-Cu(2) | 119.4(4) |
| C(19)-N(4)-Cu(2) | 118.5(4) | C(18)-N(4)-Cu(2) | 123.5(4) |
| C(21)-O(2)-Cu(2) | 114.2(4) | C(21)-O(1)-Cu(1) | 175.6(5) |
| C(25)-O(6)-Cu(1) | 111.5(4) | C(24)-O(4)-Cu(2) | 129.4(5) |
| C(28)-O(9)-Cu(1) | 119.8(4) |  |  |
| Symmetry transformations used to generate equivalent atoms: #1 x,y+1,z; #2 x,y-1,z; #3 x-y,x,z+1/6; #4 y,-x+y,z-1/6. | | | |

**Table S2c**. Selected bond lengths (Å) and angles (°) for complex **2**

| Bond lengths | | | |
| --- | --- | --- | --- |
| Cu(1)-O(6) | 1.974(2) | Cu(1)-O(9) | 1.957(2) |
| Cu(1)-N(2) | 2.029(2) | Cu(1)-N(1) | 2.025(3) |
| Cu(1)-O(2) | 2.250(2) | Cu(2)-O(4) | 1.960(2) |
| Cu(2)-O(1) | 1.966(2) | Cu(2)-N(4) | 2.013(3) |
| Cu(2)-N(3) | 2.023(3) | Cu(2)-O(11) | 2.331(3) |
| Bond angles | | | |
| O(9)-Cu(1)-O(6) | 177.57(9) | O(9)-Cu(1)-N(1) | 89.14(10) |
| O(6)-Cu(1)-N(1) | 92.00(10) | O(9)-Cu(1)-N(2) | 91.31(10) |
| O(6)-Cu(1)-N(2) | 87.45(10) | N(1)-Cu(1)-N(2) | 177.16(11) |
| O(9)-Cu(1)-O(2) | 90.47(9) | O(6)-Cu(1)-O(2) | 91.67(9) |
| N(1)-Cu(1)-O(2) | 89.98(10) | N(2)-Cu(1)-O(2) | 92.82(10) |
| O(4)-Cu(2)-O(1) | 174.57(9) | O(4)-Cu(2)-N(4) | 92.96(10) |
| O(1)-Cu(2)-N(4) | 92.04(10) | O(4)-Cu(2)-N(3) | 86.84(10) |
| O(1)-Cu(2)-N(3) | 88.57(10) | N(4)-Cu(2)-N(3) | 170.68(12) |
| O(4)-Cu(2)-O(11) | 91.04(10) | O(1)-Cu(2)-O(11) | 87.01(9) |
| N(4)-Cu(2)-O(11) | 88.10(11) | N(3)-Cu(2)-O(11) | 101.22(11) |
| C(1)-N(1)-Cu(1) | 125.3(2) | C(5)-N(1)-Cu(1) | 117.08(19) |
| C(9)-N(2)-Cu(1) | 121.9(2) | C(8)-N(2)-Cu(1) | 120.0(2) |
| C(11)-N(3)-Cu(2) | 122.1(2) | C(15)-N(3)-Cu(2) | 120.1(2) |
| C(18)#2-N(4)-Cu(2) | 122.9(2) | C(19)-N(4)-Cu(2) | 119.2(2) |
| C(21)-O(1)-Cu(2) | 114.7(2) | C(21)-O(2)-Cu(1) | 173.2(2) |
| C(24)-O(4)-Cu(2) | 129.3(2) | C(25)-O(6)-Cu(1) | 109.24(19) |
| C(28)-O(9)-Cu(1) | 120.8(2) |  |  |
| Symmetry transformations used to generate equivalent atoms: #1 x,y-1,z; #2 x,y+1,z; #3 x-y+1,x+1,z-1/6; #4 y-1,-x+y,z+1/6. | | | |

**Table S2d**. Selected bond lengths (Å) and angles (°) for complex **3**

| Bond lengths | | | |
| --- | --- | --- | --- |
| Cu(1)-O(5) | 1.948(3) | Cu(1)-N(1) | 2.014(3) |
| Cu(1)-O(1) | 2.406(6) | Cu(2)-O(2) | 1.953(3) |
| Cu(2)-N(2) | 2.016(3) | Cu(2)-O(6) | 2.408(6) |
| Bond angles | | | |
| O(5)#1-Cu(1)-O(5) | 180.0 | O(5)#1-Cu(1)-N(1) | 91.33(12) |
| O(5)-Cu(1)-N(1) | 88.67(12) | N(1)-Cu(1)-N(1)#1 | 180.0(3) |
| N(1)-Cu(1)-O(1) | 93.21(18) | N(1)#1-Cu(1)-O(1) | 86.79(18) |
| O(2)#2-Cu(2)-O(2) | 180.0 | O(2)-Cu(2)-N(2) | 91.20(12) |
| O(2)-Cu(2)-N(2)#2 | 88.80(12) | N(2)#2-Cu(2)-N(2) | 180.0 |
| O(2)-Cu(2)-O(6) | 94.75(16) | O(2)#2-Cu(2)-O(6) | 85.25(16) |
| N(2)-Cu(2)-O(6) | 87.06(18) | N(2)#2-Cu(2)-O(6) | 92.94(18) |
| C(6)-O(5)-Cu(1) | 121.0(3) | C(10)-N(1)-Cu(1) | 121.5(5) |
| C(12)-N(1)-Cu(1) | 124.3(4) | C(9)-N(1)-Cu(1) | 118.1(4) |
| C(11)-N(1)-Cu(1) | 121.4(4) | C(19)-N(2)-Cu(2) | 119.5(5) |
| C(18)-N(2)-Cu(2) | 123.6(4) | C(20)-N(2)-Cu(2) | 118.0(3) |
| C(21)-N(2)-Cu(2) | 122.2(4) | C(1)-O(1)-Cu(1) | 144.4(6) |
| C(6)-O(6)-Cu(2) | 144.6(5) |  |  |
| Symmetry transformations used to generate equivalent atoms: #1 -x+1,-y+1,-z+1; #2 -x+1,-y+2,-z+1; #3 -x+1,-y+1,-z; #4 -x+1,-y+2,-z. | | | |

**Graphs**


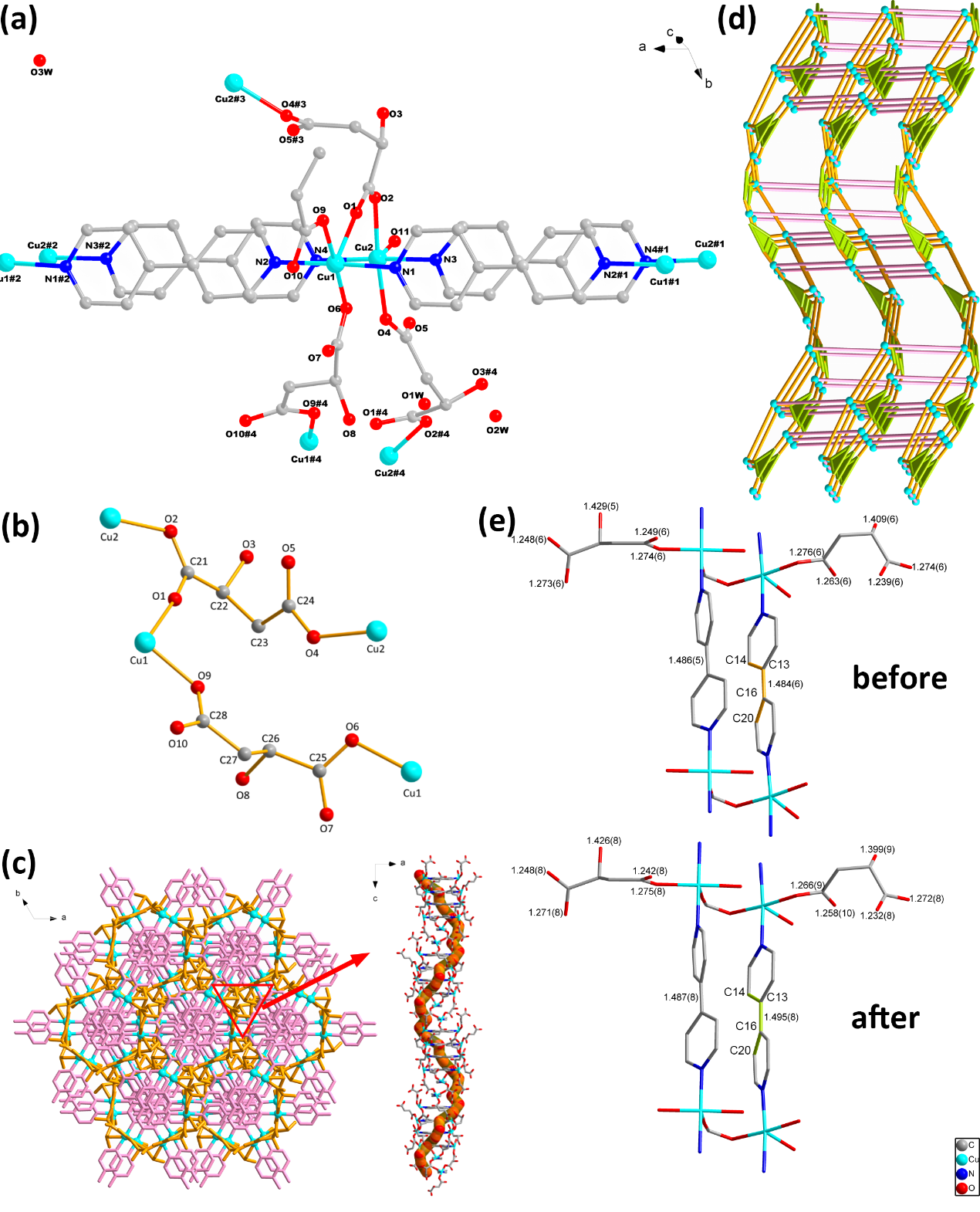


**Figure S1** Structure of **1**. (a) Coordination environments of the metal ion in **1** (H atoms are omitted). Symmetry code: #1 x-1, y, z; #2 x+1, y, z; #3 x-y, x-1, z+1/6; #4 y+1, -x+y+1, z-1/6. (b) Coordination modes of malate ions. (c) Projection of **1** on the *ab* plane and 1D-chiral chanels at *c* axis. (d) (5-c)(4-c)(3-c) topological framework of **1**. (e) Structure variation upon irradiation.


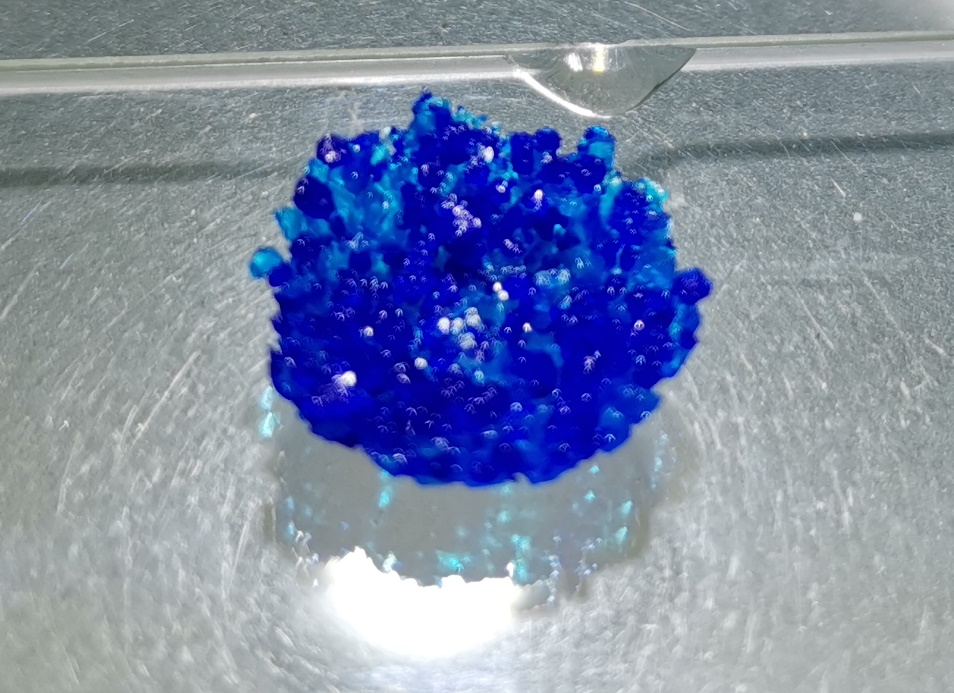


**Figure S2** Symbiosis of Cu(DL-Hmal)_2_(H_2_O)_2_ (light blue) and **3** (blue).


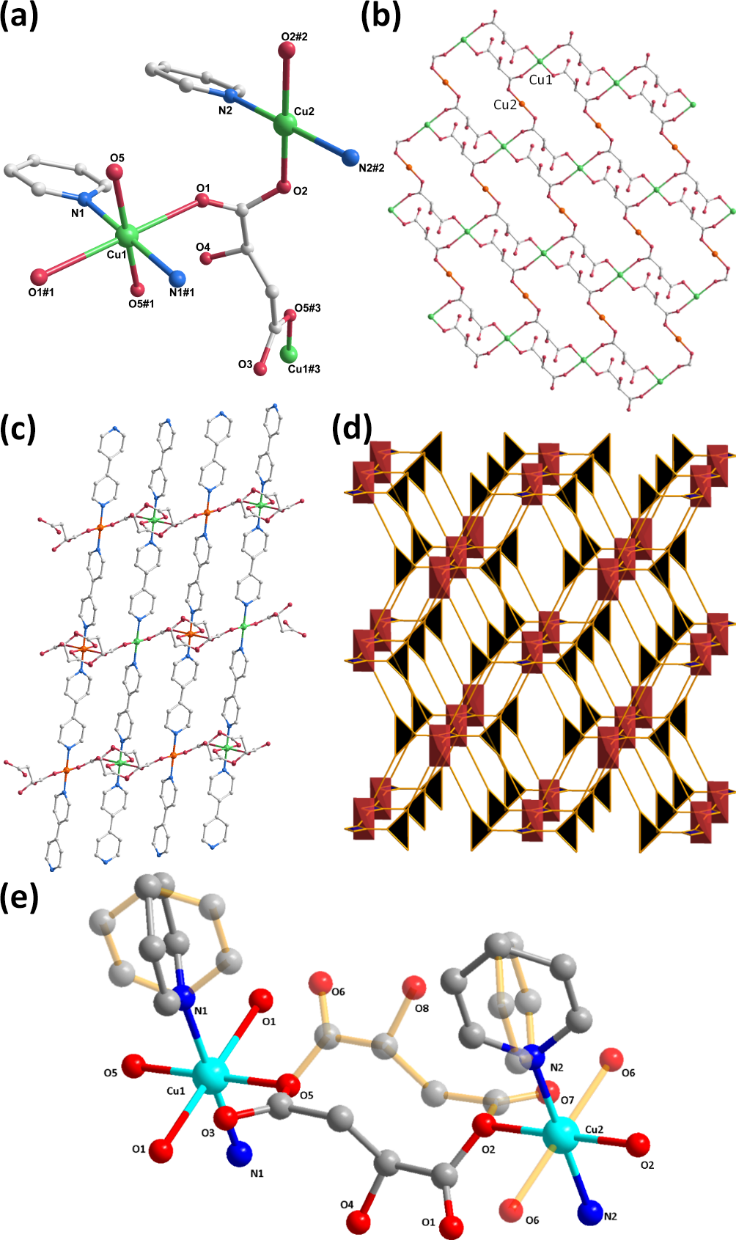


**Figure S3** Structure of **3**. H atoms and disordering of ligands are omitted for clarity. Symmetry code: #1 1-x, 1-y, 1-z; #2 1-x, 2-y, 1-z; #3 x+1, y, z. (a) Coordination environments of the metal ion in 3. (b) 2D layer of [Cu(DL-mal)]n. (c) View of 3 on the bc plane. (d) Topology of 3. Nodes: 6-c copper (red), 4-c copper (blue), 3-c malate (black). (e) Disordering of malate and bpy ligands in compound **3**.


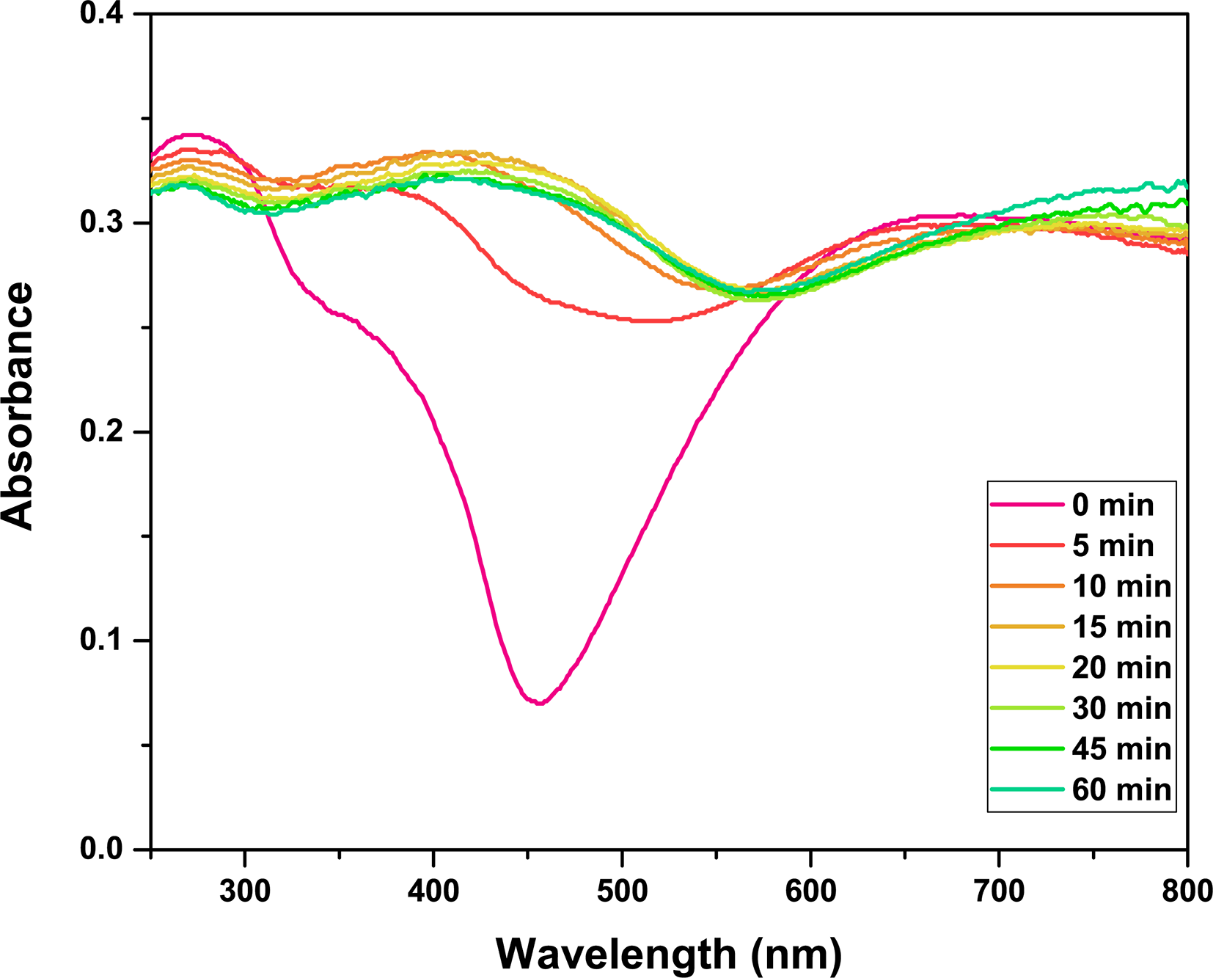


**Figure S4** UV-vis absorption spectra of **1** upon visble light irradiation.


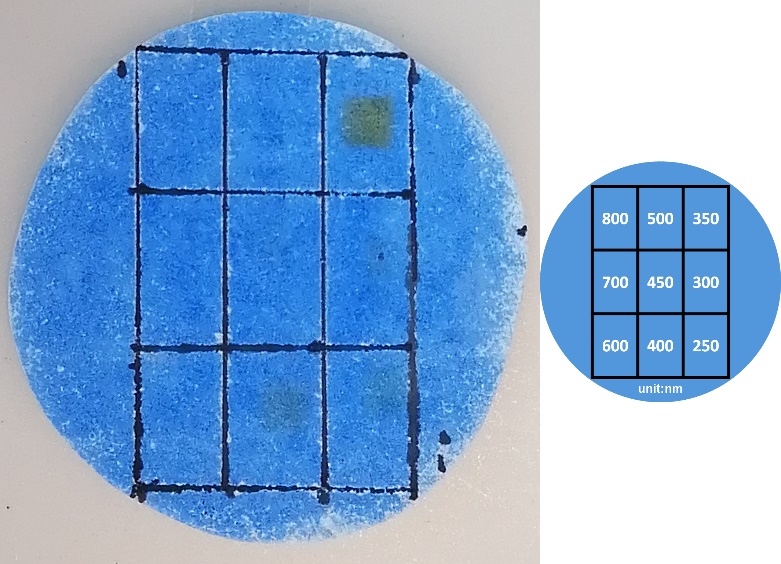


**Figure S5** Color change of **1** upon irradiation for 15 min with light at variable wavelength.


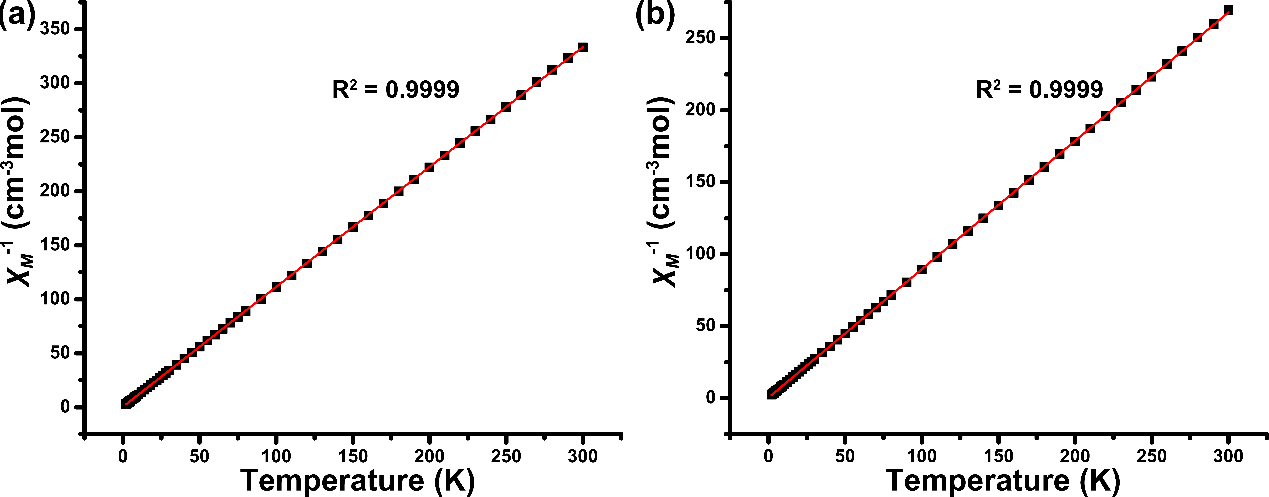


**Figure S6** Curie-Weiss Fitting of magnetic data for **1** (a) and **1P** (b).


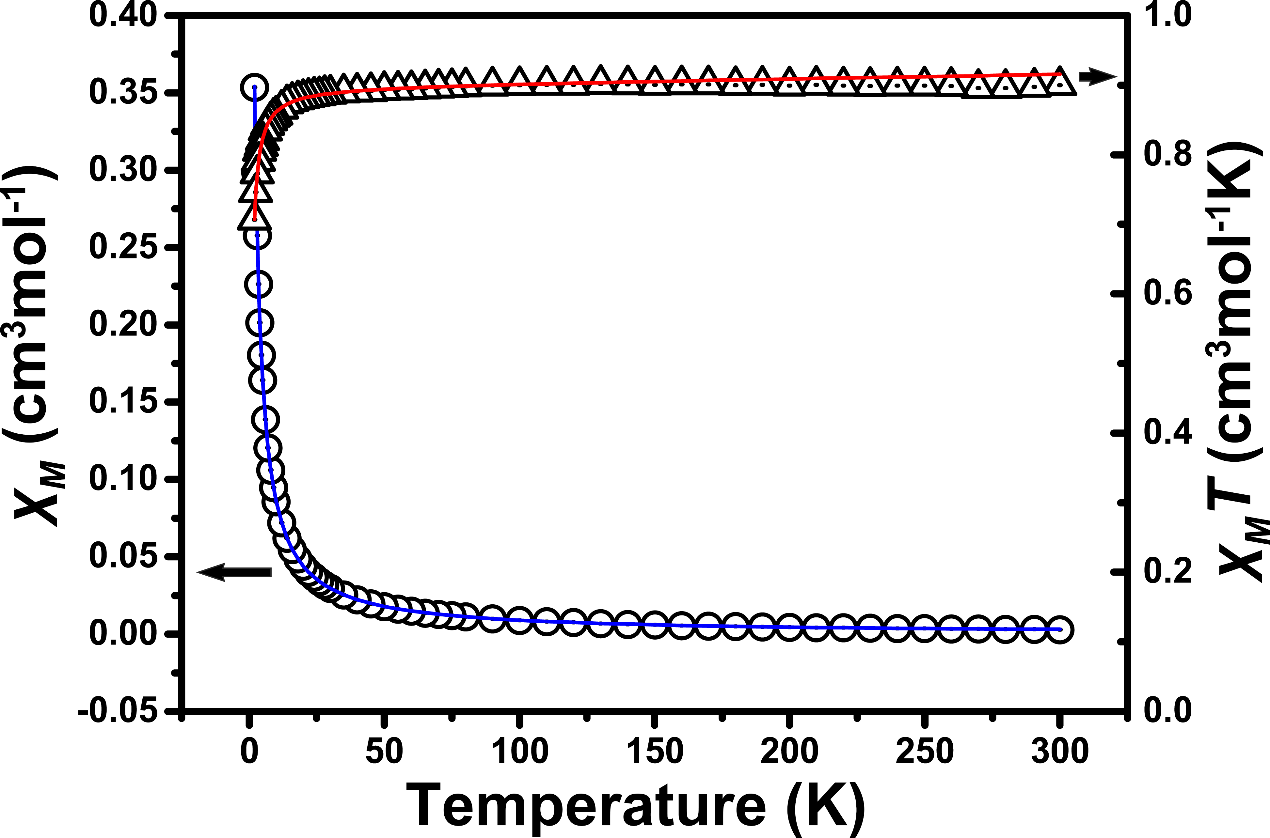


**Figure S7** Plots of *χ_M_* and *χ_M_T* versus *T* for **1**. The solid line is the best fit to the experimental data by by using the spin Hamiltonian operator.

**
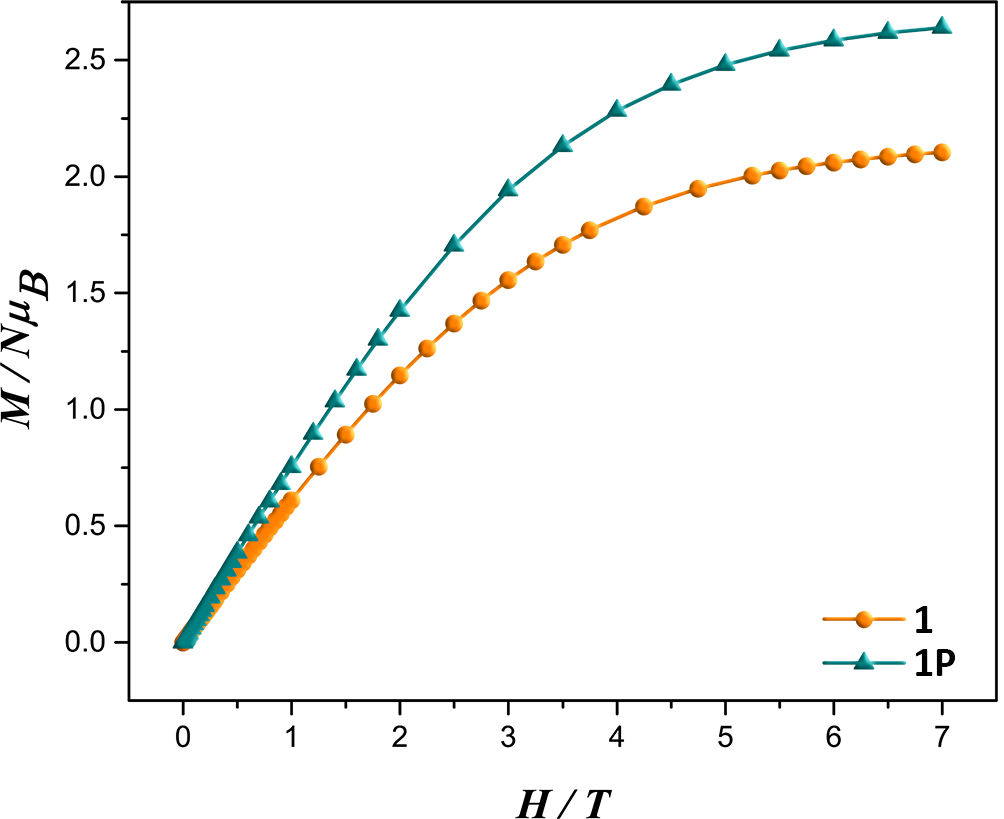
**

**Figure S8** Magnetization (M) versus ﬁeld (H) for **1** and **1P** at 2K.


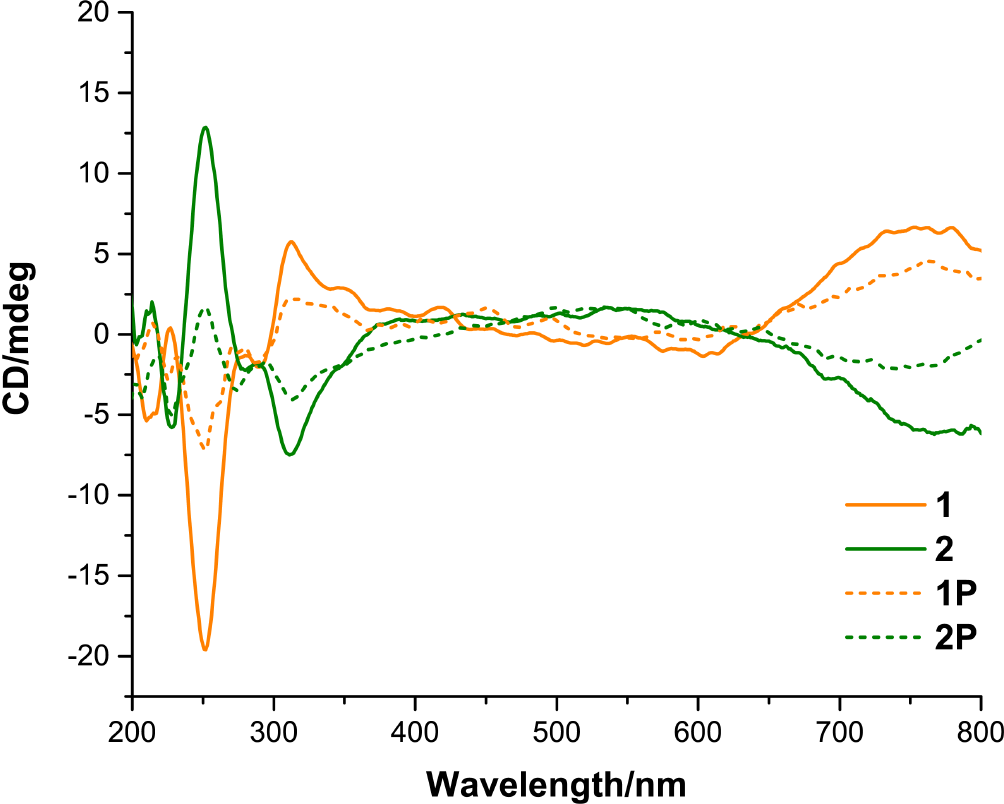


**Figure S9** Solid state CD spectra of **1**, **1P**, **2** and **2P**.


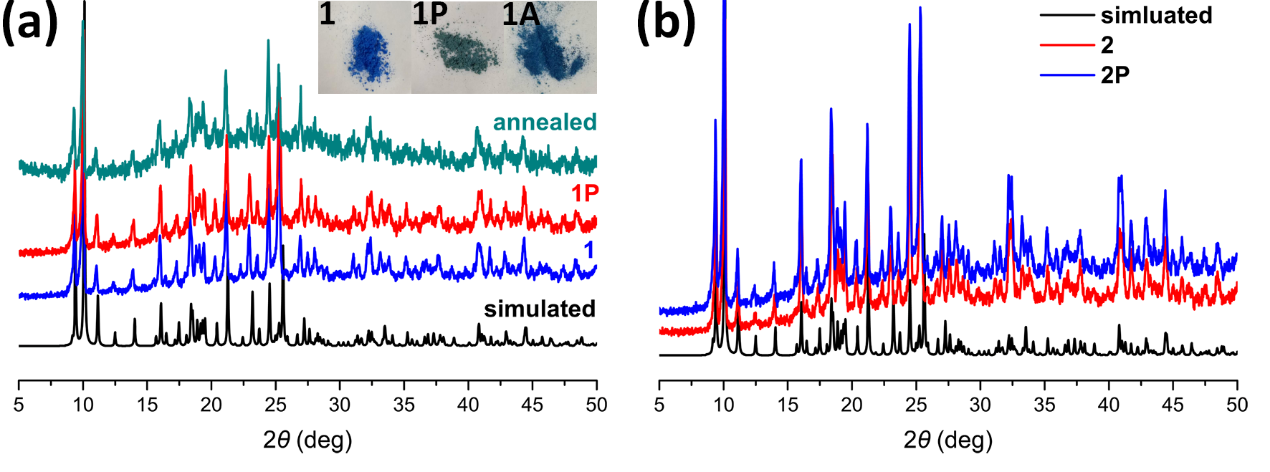


**Figure S10** PXRD of **1** and **2** before and after irradiation and annealed samples (**1A**).


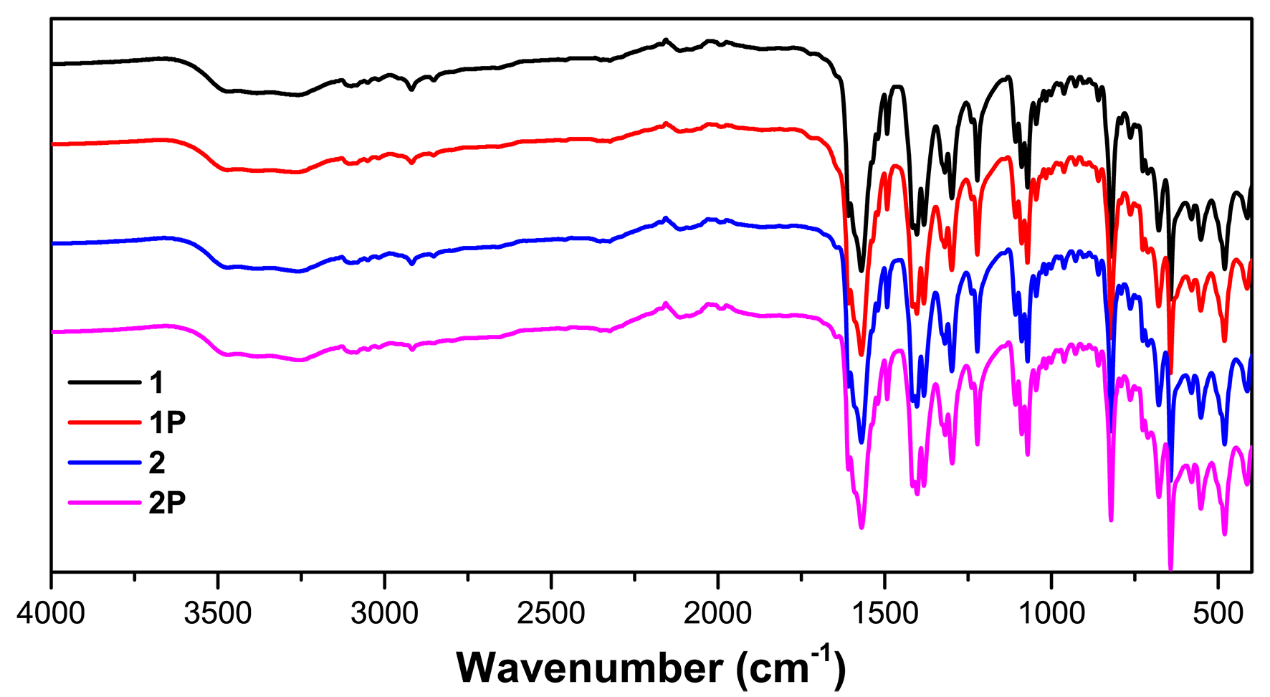


**Figure S11** FT-IR spectra of **1**, **1P**, **2** and **2P**.


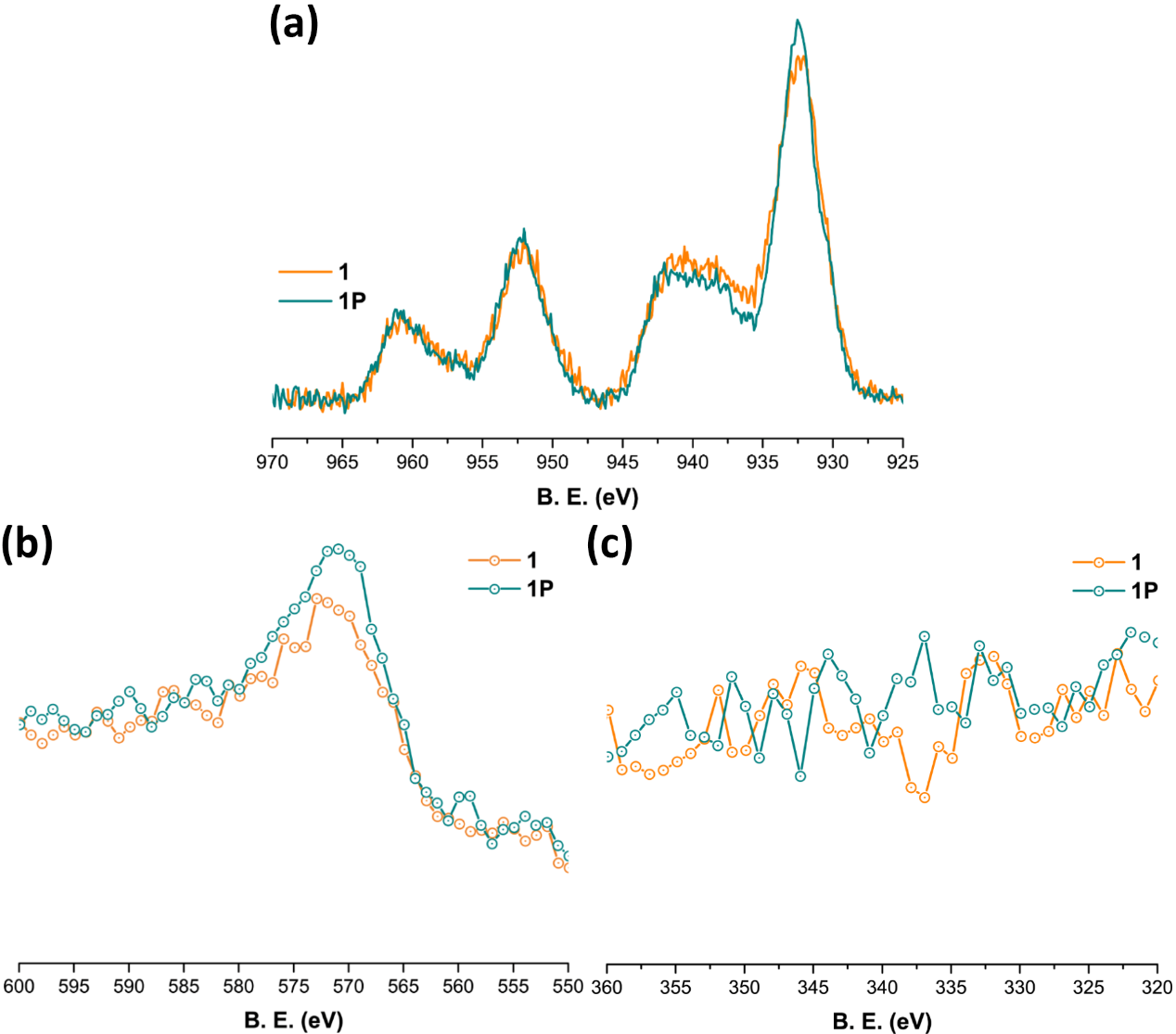


**Figure S12** Cu 2p (a), Cu (II) LLM(b) and Cu(I) LLM (c) partical XPS spectra of **1** and **1P**.


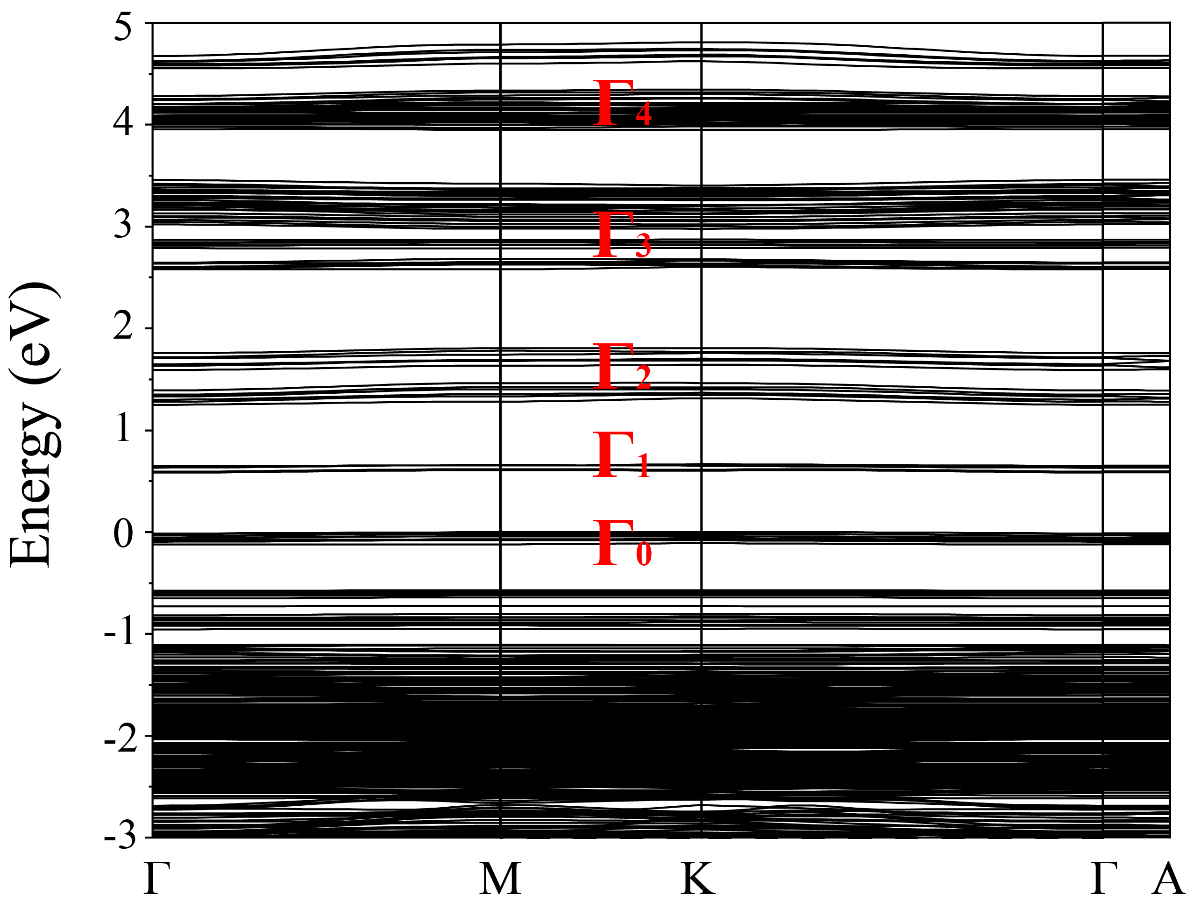


**Figure 13** Band sturcture of **1**.


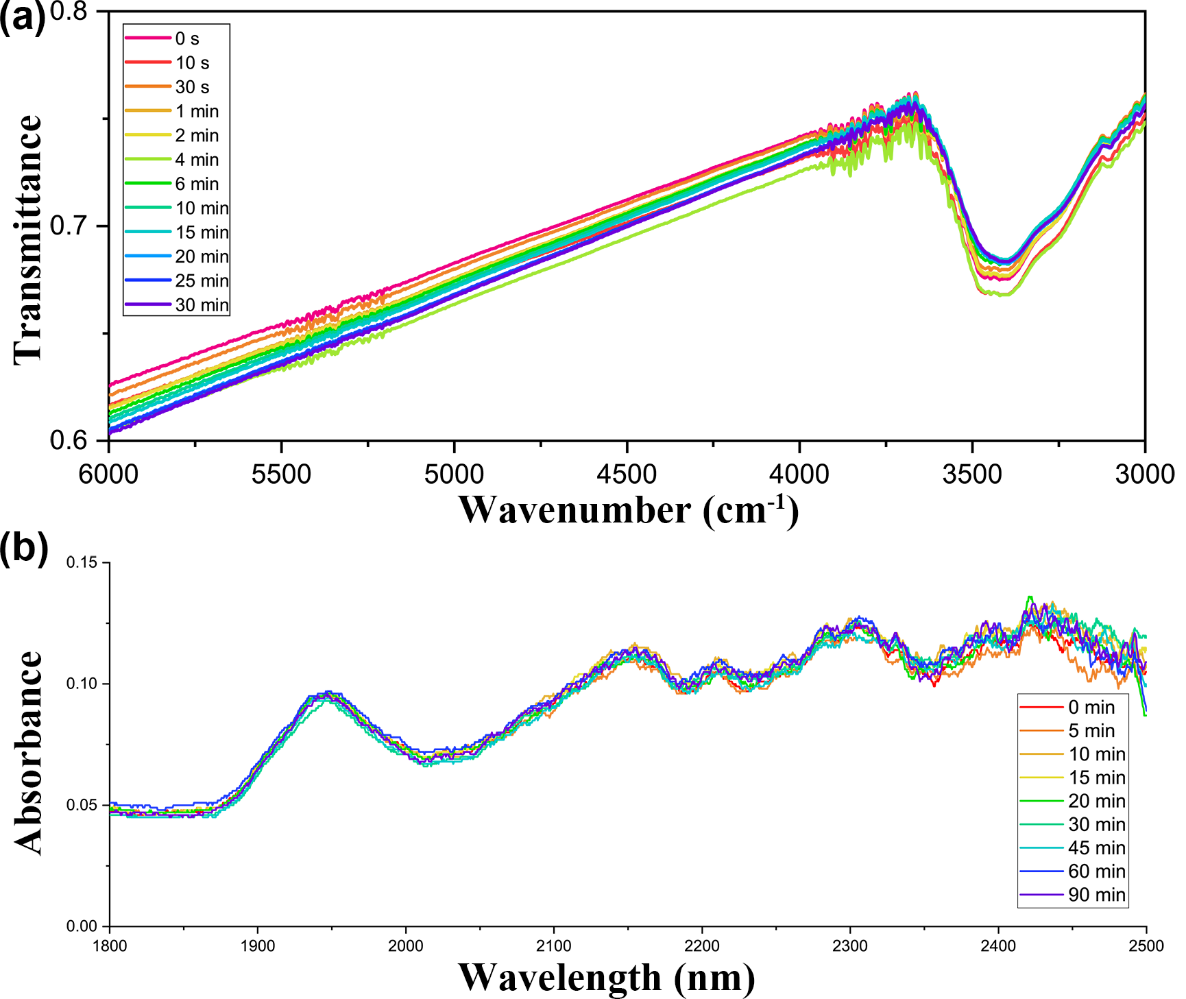


**Figure S14** Time-dependtent FT-IR spectrum of **1** with KCl Pellets (1:100) (a) and local UV-vis spectrum (b) of **1** upon UV-vis light irradiation.


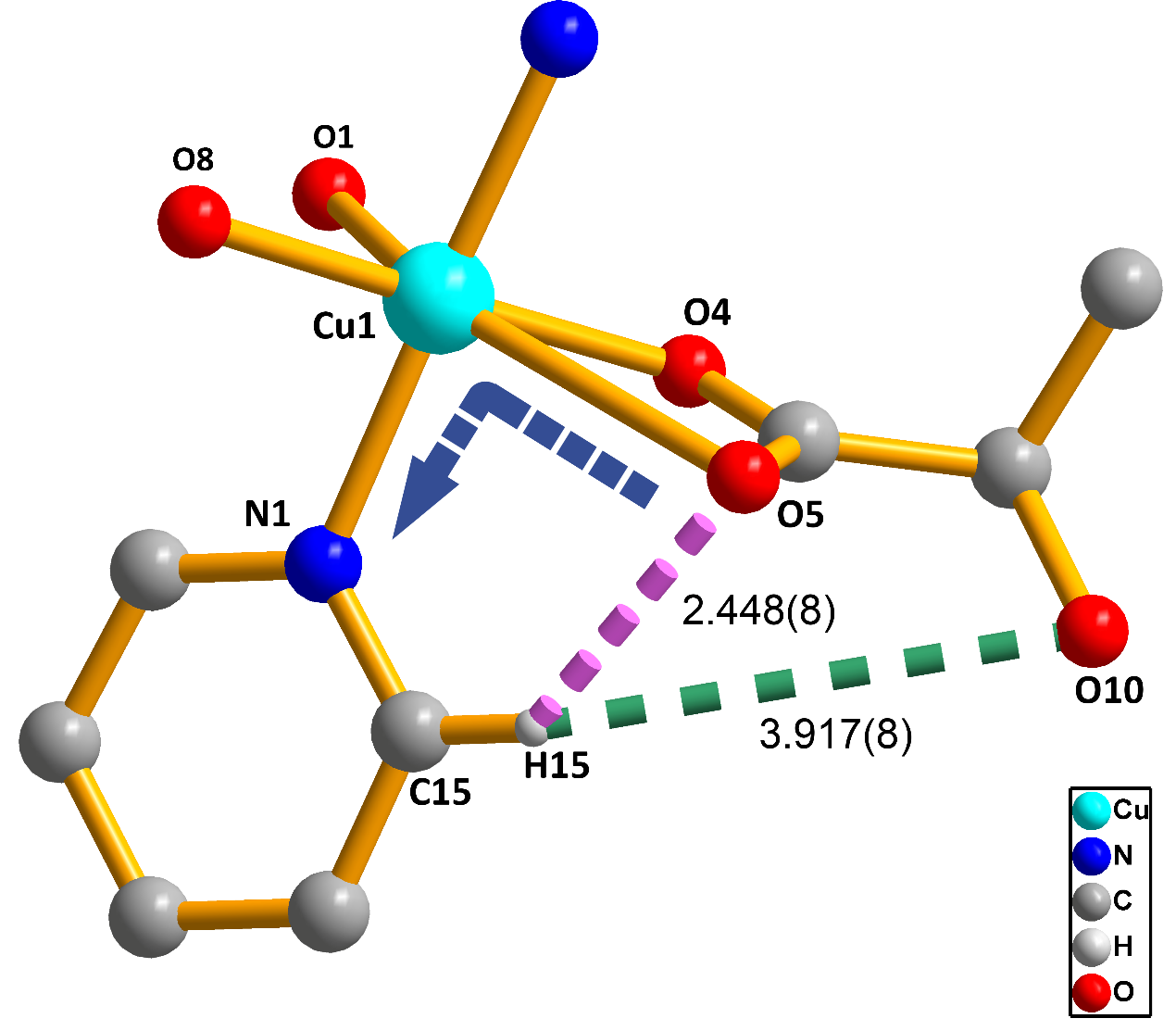


**Figure S15** Possible electron transfer passway of **1** during the photochromic process.


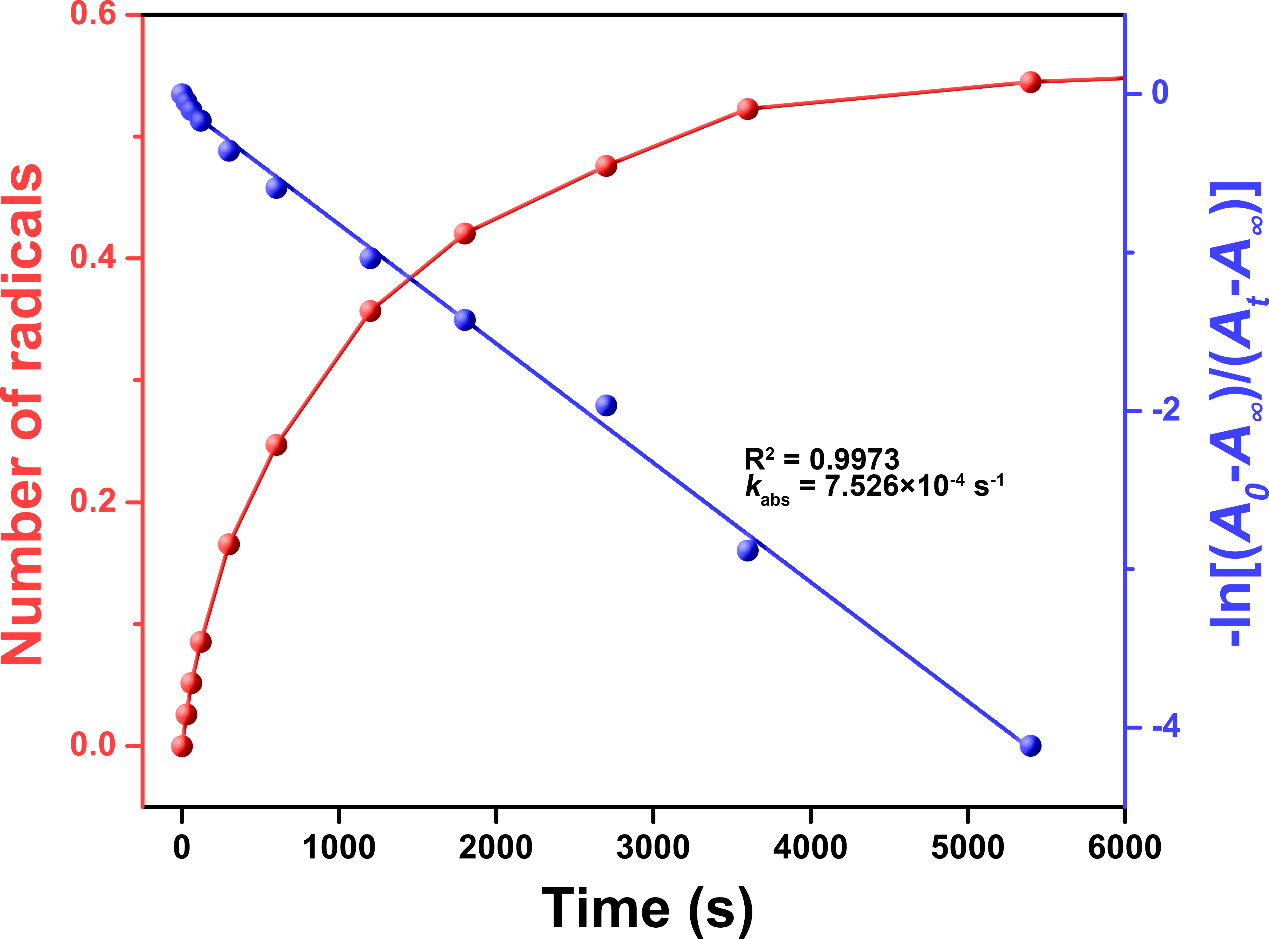


**Figure S16** Kinetics of Photoinduced electron transfer of **1**. Left: Number of photogenerated radicals (red) for a Cu_2_(L-mal)_2_(bpy)_2_(H_2_O)·3H_2_O upon irradiation. Right: First-order kinetic plot (blue) for change in absorbance at λ = 448 nm , where A_0_, A_t_, and A_∞_ are the absorbance values at time zero, time t, and infinite time of the reaction, respectively.


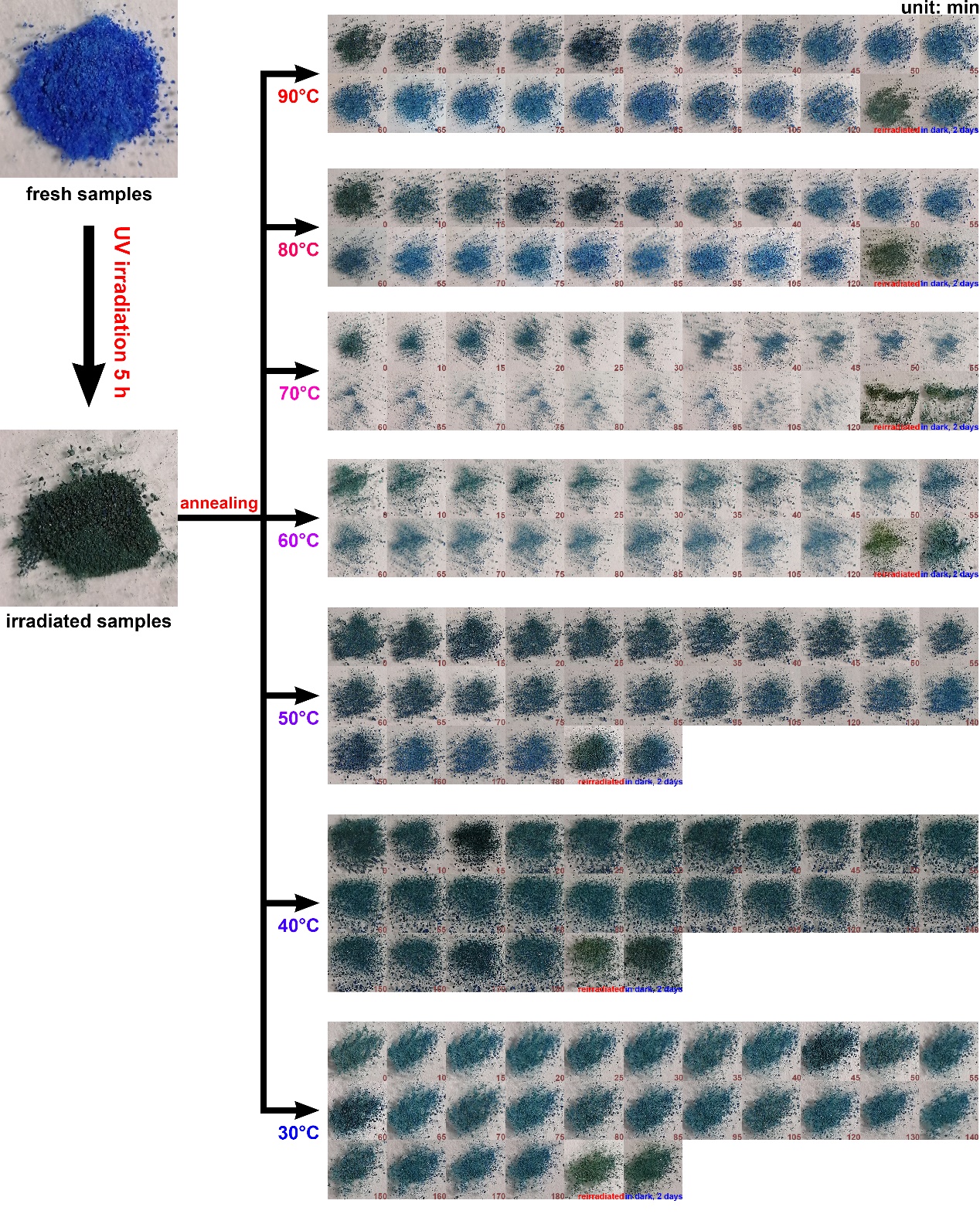


**Figure S17** Color change of **1** upon irradiation and heating at variable temperature.


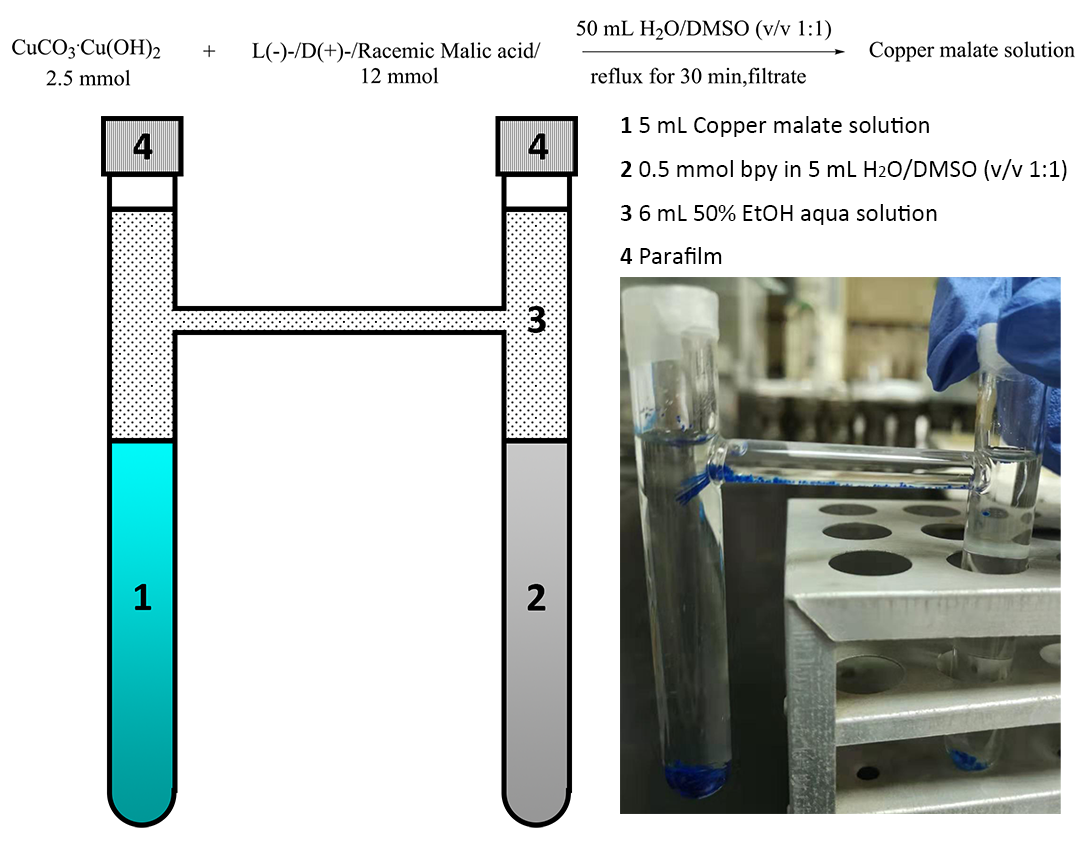


**Scheme S1** Schematic diagram of diffusion method for preparations of **1**-**3**.


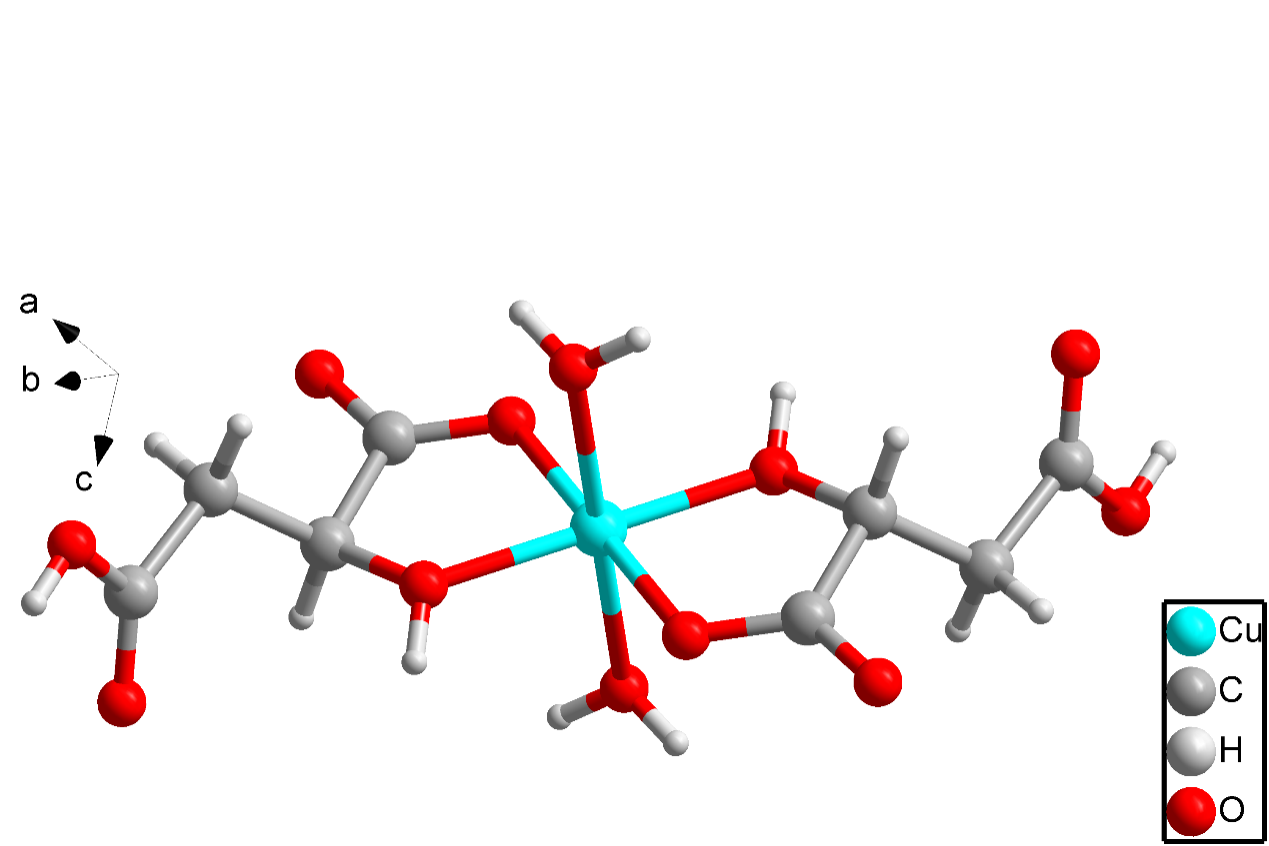


**Figure S18** Sturcture of Cu(Hmal)_2_(H_2_O)_2_.
